# Differential *O*- and Glycosphingolipid Glycosylation in Human Pancreatic Adenocarcinoma Cells With Opposite Morphology and Metastatic Behavior

**DOI:** 10.3389/fonc.2020.00732

**Published:** 2020-06-02

**Authors:** Tao Zhang, Irma van Die, Boris Tefsen, Sandra J. van Vliet, Lisa C. Laan, Jing Zhang, Peter ten Dijke, Manfred Wuhrer, Ana I. Belo

**Affiliations:** ^1^Center for Proteomics and Metabolomics, Leiden University Medical Center, Leiden, Netherlands; ^2^Department of Molecular Cell Biology and Immunology, Cancer Center Amsterdam, Amsterdam UMC, Vrije Universiteit Amsterdam, Amsterdam, Netherlands; ^3^Department of Biological Sciences, Xi'an Jiaotong-Liverpool University, Suzhou, China; ^4^Department of Cell and Chemical Biology and Oncode Institute, Leiden University Medical Center, Leiden, Netherlands

**Keywords:** glycosylation, gene array analysis, glycosyltransferase, *O*-glycosylation, glycosphingolipid (GSL) glycans, pancreatic ductal adenocarcinoma

## Abstract

Changes in the glycosylation profile of cancer cells have been strongly associated with cancer progression. To increase our insights into the role of glycosylation in human pancreatic ductal adenocarcinoma (PDAC), we performed a study on *O*-glycans and glycosphingolipid (GSL) glycans of the PDAC cell lines Pa-Tu-8988T (PaTu-T) and Pa-Tu-8988S (PaTu-S). These cell lines are derived from the same patient, but show an almost opposite phenotype, morphology and capacity to metastasize, and may thus provide an attractive model to study the role of glycosylation in progression of PDAC. Gene-array analysis revealed that 24% of the glycosylation-related genes showed a ≥ 1.5-fold difference in expression level between the two cell lines. Subsequent validation of the data by porous graphitized carbon nano-liquid chromatography coupled to a tandem ion trap mass spectrometry and flow cytometry established major differences in *O*-glycans and GSL-glycans between the cell lines, including lower levels of T and sialylated Tn (sTn) antigens, neoexpression of globosides (Gb3 and Gb4), and higher levels of gangliosides in the mesenchymal-like PaTu-T cells compared to the epithelial-like PaTu-S. In addition, PaTu-S cells demonstrated a significantly higher binding of the immune-lectins macrophage galactose-type lectin and galectin-4 compared to PaTu-T. In summary, our data provide a comprehensive and differential glycan profile of two PDAC cell lines with disparate phenotypes and metastatic behavior. This will allow approaches to modulate and monitor the glycosylation of these PDAC cell lines, which opens up avenues to study the biology and metastatic behavior of PDAC.

## Introduction

Pancreatic adenocarcinoma is an important cause of cancer-related death in the western world. Especially, pancreatic ductal adenocarcinoma (PDAC), accounting for 90% of the pancreatic adenocarcinoma, has a very poor prognosis. The 1-year survival rate is reported to be 20%, and the 5-year rate <5% for all stages of pancreatic cancer combined (1). These low survival rates are mainly due to a lack of effective diagnostic strategies, the rapid progression of pancreatic cancer at an early stage, and unreliable targets for therapeutic intervention. It is of great importance to develop reliable diagnostic biomarkers and to understand molecular and cellular mechanism in PDAC metastasis.

Glycosylation is one of the most common and complex post-translational modifications of proteins, and glycans also occur conjugated to lipids (2). Changes in glycosylation are involved in the regulation of many cancer-related processes such as signaling, cell adhesion, tumor proliferation, invasion, metastasis, and angiogenesis (2–5). Importantly, cancer-associated glycans also provide a set of diagnostic biomarkers and potential targets for therapeutic strategies (2, 5–7). For example, CA 19-9 which is an antigen carrying the glycan epitope sialyl Lewis A (sLe^A^) is a widely used biomarker in clinical diagnosis of PDAC (4, 8). The recently reported non-invasive diagnostic biomarker for early stages of pancreatic cancer glypican-1 is also a cell surface proteoglycan (9), indicating again the importance of glycosylation in PDAC. Although, many glycan changes have been observed in pancreatic cancer [reviewed in (4)], the causes as well as the consequences for cancer progression are still incompletely understood.

Many factors can contribute to changes in cancer glycosylation, including regulation and localization of the enzymes that are involved in their biosynthesis [glycosyltransferases (GTs) and glycosidases], enzymes synthesizing glycan precursors, nucleotide sugar donors and transporters, and alteration of the peptide backbone (2, 10, 11). Among them, GTs catalyze the transfer of a monosaccharide to the initiating and growing glycans, thereby showing a key role in the regulation of glycosylation and aberrant biosynthesis of core-glycans in cancer (12, 13). Importantly, changes in enzymes involved in the modification and termination like fucosyltransferases (FUTs) (14) and sialyltransferases (STs) (15, 16) have been associated with progression of invasive malignancies by increasing apoptosis resistance, affecting tumor growth, metastasis formation, or resistance to therapy.

For pancreatic cancer, several aberrations in *N*- and *O*-glycosylation and their regulation have been described Munkley (4), Taniuchi et al. (13), Radhakrishnan et al. (17), Hofmann et al. (18), and Chugh et al. (19). To further understand the expression and regulation of glycosylation in metastasis of pancreatic cancer, we performed a comprehensive glycosylation study, including *O*- and GSL-glycosylation analysis starting from differential gene expression and activity of GTs, to the structural and relative quantitative analysis of their glycan end-products, in two closely related pancreatic cell lines. These two cell lines, originate from a single liver metastasis of a PDAC (20) and display disparate grades of differentiation and have distinct properties that are relevant for PDAC progression. Pa-Tu-8988S (PaTu-S) cells exhibit an epithelial-like phenotype, with polarity, E-cadherin expression, preserved cell-cell contacts, and low migratory properties. Pa-Tu-8988T (PaTu-T) cells are spindle-like with increased cell projections and minimal cell-cell contacts (21). They have a more mesenchymal phenotype and are highly migratory *in vitro*. Most remarkable is the much stronger metastatic potential of PaTu-T, compared to PaTu-S cells in a zebrafish model (21–23). Therefore, these sister cell lines are an attractive model to study the role of glycosylation in the cell biology of human PDAC.

Using glyco-gene microarray analysis we show that a quarter of the ~1,171 glycosylation-related genes displayed differential expression in PaTu-S and PaTu-T. A large variation in the *N*-glycosylation signature between these two cell lines has been previously reported (24). Here, we report the analysis of their *O*- and GSL-glycosylation patterns, and demonstrate divergent abundance of glycan structures and binding to lectins between the cell lines using porous graphitized carbon nano-liquid chromatography coupled to a tandem mass spectrometer (PGC nano-LC-ESI-MS/MS) and flow cytometry, respectively. In addition, we show that both PDAC cell lines differentially interact with immune-related lectins, likely as a consequence of the dissimilarity in surface glycosylation, which may result in their differential recognition by innate immune cells.

## Results

### Gene Expression Profiling

**Figure 1:**
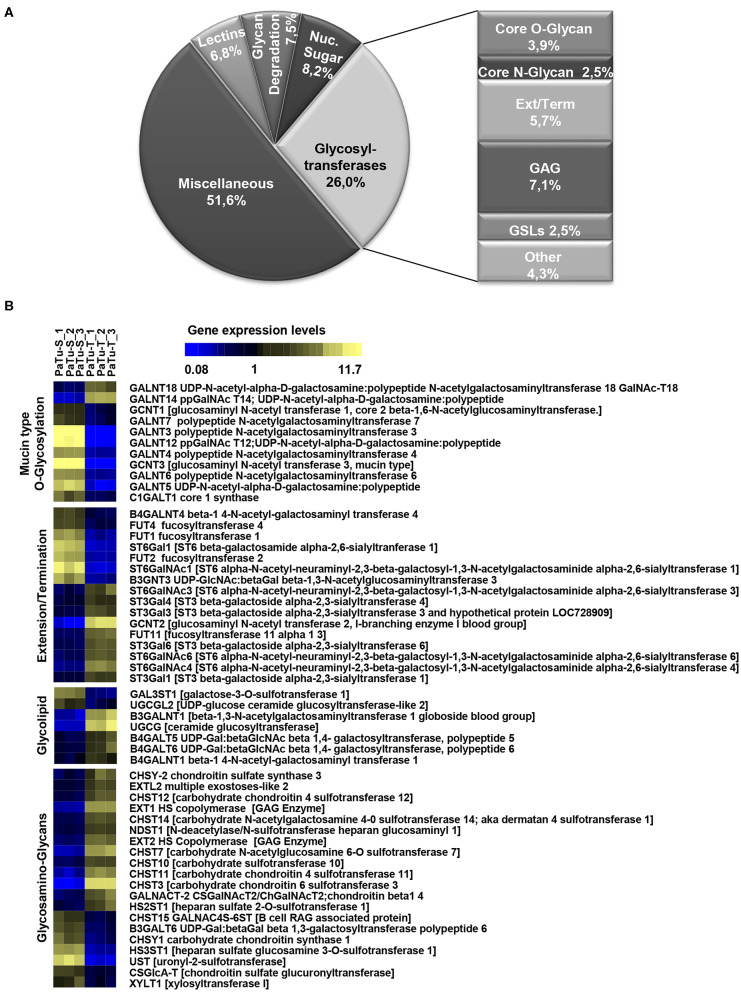
Expression levels of glycosylation-related genes in PaTu-T and PaTu-S cell lines. **(A)** Glyco-gene transcripts with more than 1.5-fold difference in transcription levels between the two cell lines (281 from a total of 1,171 gene transcripts) clustered according to their putative function. The miscellaneous group includes genes related to glycosylation such as growth factors, receptors, interleukins, and adhesion molecules. Glycosyltransferases (GTs) comprises 73 of the 281 genes (26%) and are classified according to their assumed role in biosynthesis of target structures like *O*-glycan core, *N*-glycan core, GAG (glycosaminoglycan), GSLs (glycosphingolipids), Term (terminal modifications of various types of glycan structures), and Ext (extension of the various types of glycan structures). **(B)** TreeView of GT gene expression levels in PaTu-S and PaTu-T. Expression levels are depicted from low expressed genes (dark blue), through middle range gene expression (black) to highly expressed genes (yellow).

To determine the differential glycosylation potential of the PaTu-S and PaTu-T cell lines, we analyzed the expression profiles of glycosylation-related genes in these cells via Affymetrix microarray analysis, and selected for genes that showed a significant ≥1.5-fold difference in expression levels between the two cell lines (*P* < 0.001). Remarkably, 281 genes (24% of all genes within the array), met this criterion (Figure 1A). The largest group of these 281 differentially expressed genes consists of GT genes (26%), indicating a major difference in glycan biosynthetic potential between PaTu-T and PaTu-S. A pathway analysis was performed manually by grouping these GTs in known glycosylation pathways and subsequently a Treeview analysis was generated utilizing selected groups of GT genes (Figure 1B).

The observed changes in expression levels of 7 genes involved in core *N*-glycosylation have been described previously in detail (24). In the GAG gene family, 20 gene transcripts displayed a more than 1.5-fold difference in expression between the two cell lines, and most of them were higher expressed in PaTu-T (Figure 1B). The enzymes encoded by these genes are almost exclusively involved in the synthesis of heparan sulfate (HS) or chondroitin sulfate (CS). The important role of expression of HS and CS and their sulfation on proteoglycans in cancer metastasis has been demonstrated previously (6). Remarkably, whereas two transcripts that encode enzymes (*XYLT1* and *B3GALT6*, Figure 1B) involved in synthesis of the common core for HS and CS were higher expressed in PaTu-S, genes for extension of HS, and in particular sulfation in CS, were mostly higher expressed in PaTu-T.

**Figure 2:**
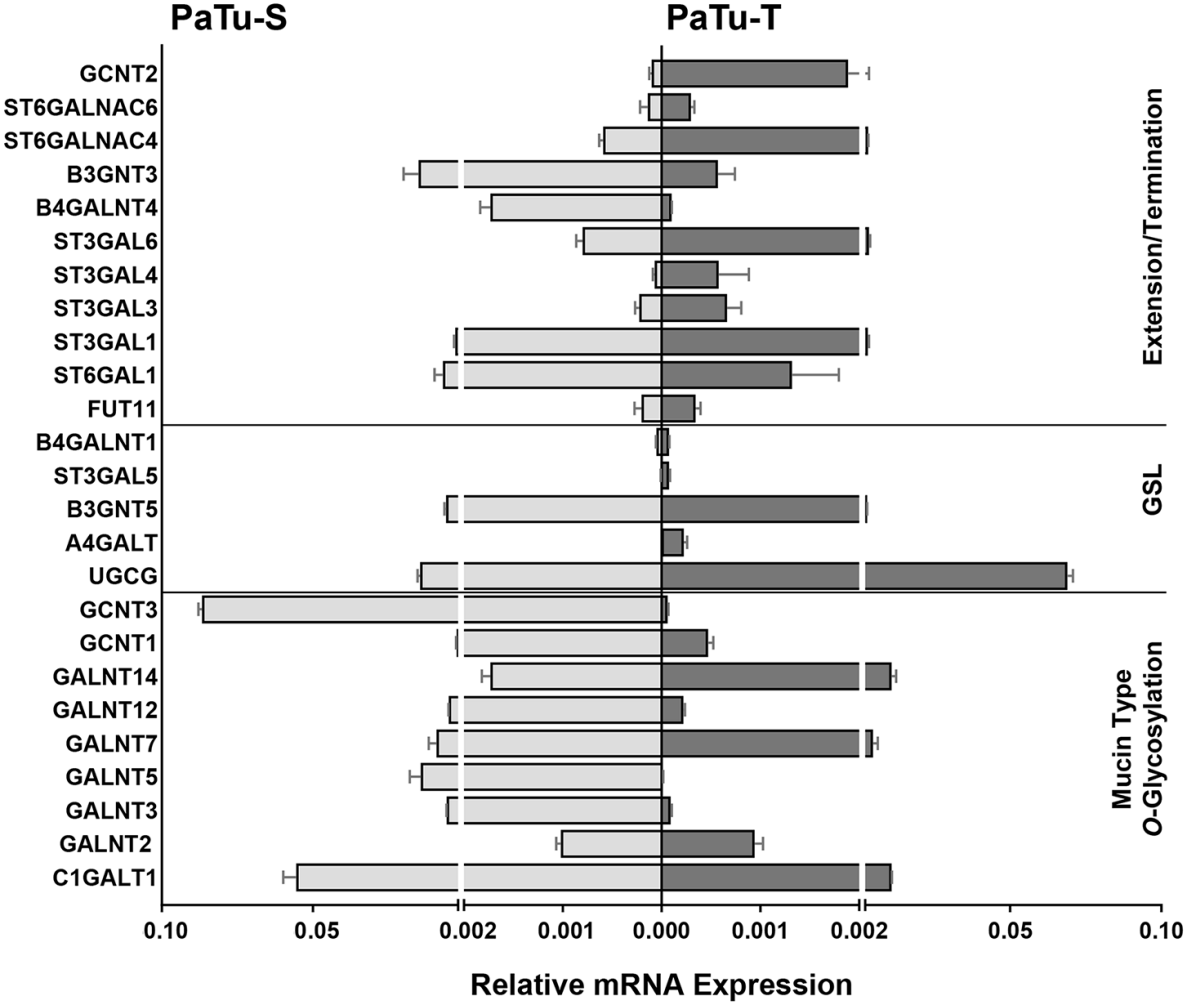
mRNA levels of selected GT genes in PaTu-S and PaTu-T. Validation by qRT-PCR of the expression of selected GT genes involved in *O*-glycan core and glycosphingolipid synthesis as well as in extension or termination of various glycan structures in PaTu-S (light gray bars) vs. PaTu-T cells (dark gray bars). mRNA levels are shown as relative abundance to the household reference gene GAPDH ± SEM of at least 3 independent experiments.

Of the differentially expressed GT genes, 11 are putatively involved in mucin-type core *O*-glycosylation. Many of these gene transcripts were higher expressed in PaTu-S compared to PaTu-T cells, including several *ppGALNTs*, enzymes that catalyze the addition of GalNAc to Ser or Thr residues in glycoproteins. Most results observed in the gene array were confirmed by quantitative real-time PCR (Figure 2) using a set of specific primers (Table 1). Remarkably, *GALNT3, GALNT5*, and *GALNT12* are almost exclusively expressed in PaTu-S, whereas *GALNT14* was 6 times higher expressed in PaTu-T (Figure 2). Furthermore, *C1GALT1*, encoding the core 1 enzyme and *GCNT3* coding for the core 2/4 enzyme were expressed 5-fold and >1000-fold higher in PaTu-S compared to PaTu-T. No significant difference was observed in expression levels of *GALNT2*, which showed no differential expression between PaTu-T and -S in the gene array and was included as a control. In summary, our data indicate that the two cell lines differ profoundly in their potential to synthesize mucin-type *O*-glycans.

**Table 1:** Sequences of primers used in this study for quantitative Real-Time PCR.

**GT name**	**Forward (5′ to 3′)**	**Reverse (5′ to 3′)**	**References**
*GAPDH*	CCATGTTCGTCATGGGTGTG	GGTGCTAAGCAGTTGGTGGTG	(78)
*A4GALT*	AACACTGGGGTGATGCAGG	AAATCCATCTCGATATTCTGCAGTG	This study
*B3GNT3*	GTGGGACTTCCACGACTCCTT	GCACCTTGTCTCCTGCCACT	(78)
*B3GNT5*	CCAGCGACTTTAGCTCCGAT	TTCCATGCCACCTCCAAGTC	This study
*B4GALNT1*	GCGTTAGACAGGATGTGGCTGGG	GCCCACGGCGCAAGAGGTAG	This study
*B4GALNT2*	CTGGCTGCCCTAGAGAAGACC	CACCTTTATGCGGCACATTG	(78)
*B4GALNT4*	TGGTCAAGGACTTCCCGATC	AGGCGAGTGTAGTCGTTGGG	(78)
*B4GALT1*	AACTTGACCTCGGTCCCAGTGC	GGCCGCCCATCTTCACATTTG	(78)
*C1GALT1*	CATCCCTTTGTGCCAGAACACC	GCAAGATCAGAGCAGCAACCAG	(78)
*C1GALT1C1*	CACCACCATGAGCATCATCAC	CATGCGCTCATCCTCTGAAA	(78)
*FUT11*	GCGCCACATCCCGGTAGACT	TGTCCTGTAGCCGCGCGGTA	This study
*GALNT2*	GCTTCTGTTGGAGGGACGAT	CCACCTGGTTGAACTTGTTG	Glycogene
*GALNT3*	CGACAGCAACCGGAGTCGCA	ATGGTAGTACCTGGCGGGTGGGC	This study
*GALNT5*	CTCCAGGGCAGTTTGGGCGTC	GCTGCTCTGCACATCCAGCAGGT	This study
*GALNT7*	GCCCGAGGAGCATGGGATTGG	GCTGGGGACCGATACGGTTCAG	This study
*GALNT12*	CAAGAGAGAGGGCCTGGTG	CCAGGAAGGTCAGAACATCG	Glycogene
*GALNT14*	CAAGGAAATCGTCGTCAACC	AGAGCTCACCATGTCCCAGT	Glycogene
*GCNT1*	GCCGCAGTGTCACCTTCCACA	TCTGGTCCCAGGAAACCTCAAAGA	This study
*GCNT2*	ACAGCGTTGAAACCGCCTC	TCATGCAGAACAAAGTTGGCA	This study
*GCNT3*	GCTGGTCAAGTGGCAGGGT	TGGCCAACAGGTGATGGTT	(78)
*ST3GAL1*	GGGCAGACAGCAAAGGGAA	GGCCGTCACGTTAGACTCAAA	(78)
*ST3GAL3*	GGGTCACGAATTGACGACTATG	GTGATGCGCAGTGTCGTTTT	(78)
*ST3GAL4*	TCGAGCGATGGTTTCTCCCACAA	CCGGGAGTAGTTGCCAAAGAGC	This study
*ST3GAL6*	GGCCATATTCCTGAGTGCTGTC	AGCTGGCTTTGATAAACAAGGC	This study
*ST3GAL5*	GCACCACTGTCTGACCTTGA	CCAGAATGGCAGGGTTTCCT	This study
*ST6GAL1*	CATCCAAGCGCAAGACTGACG	TGTGCCCTGGTTGAGATGCTTC	(78)
*ST6GALNAC4*	GTTCACCATGATCCTCGCG	TGACACTCATCTAGCCGGCC	(78)
*ST6GALNAC6*	TCGAGCGATGGTTTCTCCCACAA	CTCGGGCCACCTTTGGGTCCT	This study

The array data showed differential expression of 7 GT genes that are involved in GSLs biosynthesis. Interestingly, the UDP-glucose ceramide glucosyltransferase (*UGCG*) transcript which catalyzes the first glycosylation step in GSL-biosynthesis showed 5 times higher expression in PaTu-T compared to PaTu-S (Figure 2). The gene expression of four key enzymes responsible for the synthesis of globosides, gangliosides, and (neo)lacto-series GSLs (nsGSLs), namely *A4GALT, ST3GAL5, B4GALNT1*, and *B3GNT5*, was also analyzed (Figure 2). It revealed a neoexpression of *A4GALT*, a 9-fold higher level of *ST3GAL5* and a 1.5-fold higher expression of *B4GALNT1* in PaTu-T cells. However, a 2-fold lower expression of *B3GNT5* was observed. These different expression levels may lead to specific expression of globosides, elevated expression of gangliosides, and a decreased level of nsGSLs in PaTu-T cells, respectively.

Furthermore, gene transcripts involved in the extension and termination of the core structures of *N*-glycans, *O*-glycans, or GSLs, were differentially expressed between PaTu-S and PaTu-T (Figures 1, 2). Examples are *GCNT2*, which was 19 times higher in PaTu-T and which is involved in the extension of type 2 glycans and glucosylceramide-based GSLs. By contrast, *B3GNT3*, involved in polylactosamine synthesis, was higher expressed in PaTu-S. Transcripts involved in terminal reactions of different glycan-types, which were higher in PaTu-S compared to PaTu-T include *FUT1* and *FUT2*. These results are consistent with our previous findings on the elevated expression of *FUT1* and *FUT2* regulated by hypoxia inducible factor α (25). In addition, PaTu-S cells displayed higher levels of *ST6GAL1* (5.3 FC), which suggests an elevated capacity for the α2,6 sialylation of terminal galactose. Similarly, the expression of *ST6GALNAC1*, which can sialylate Tn antigens to form the cancer-associated sialylated Tn antigen (sTn) (26), was higher in PaTu-S compared to PaTu-T (Figure 1B). By contrast, PaTu-T cells show an increased potential for α2,3 sialylation encoded by ST genes *ST3GAL3* (3.0 FC)*, ST3GAL4* (8.2 FC), and *ST3GAL6* (4.2 FC). Also, expression levels of *ST6GALNAC4* (5.1 FC) and *ST6GALNAC6* (2.2 FC), involved in sialylation of core 1 and 2 *O*-glycans, globosides, and gangliosides were more than 2 fold higher in PaTu-T.

In summary, based on the observed GT expression data our findings suggest that PaTu-T and PaTu-S display major differences in cellular glycosylation.

### Biosynthesis of *O*-Glycans

**Figure 3:**
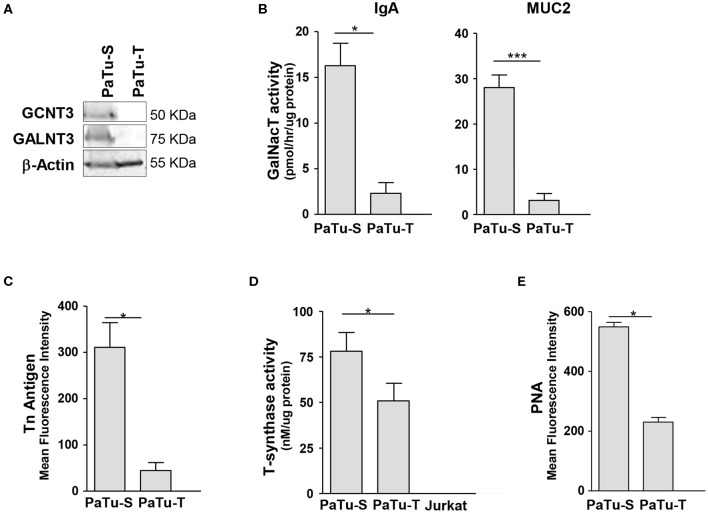
Biosynthesis of *O*-glycan structures. **(A)** Protein levels of GCNT3 and GALNT3 in PaTu-S and PaTu-T. Total proteins (150 μg) from whole-cell extracts were analyzed by Western blotting using anti-GCNT3 and anti-GALNT3 for detection of the respective proteins and anti-actin as the protein loading control. **(B)** ppGALNT activity was determined using whole cell lysates from PaTu-S and PaTu-T cells as enzyme source, UDP-[^3^H]-GalNAc as a donor, and MUC2 and IgA hinge region peptide as acceptor substrates. Radioactivity incorporated in the peptide-products was determined. **(C)** Binding of anti-Tn mAb to PaTu-S and PaTu-T cells as measured by flow cytometry, and shown as average MFI ± SEM of at least 3 independent experiments. **(D)** T-synthase activity was measured using whole cell lysates from PaTu-S, PaTu-T, and Jurkat cells (as negative control) as enzyme sources, UDP-Gal as a donor and GalNAc-α-(4-MU) as acceptor. Final product was measured by fluorescence (excitation 360 nm and emission at 460 nm). Results are given as average enzyme activity ± SEM of at least 3 independent experiments. **(E)** Binding of PNA recognizing terminal Galβ1-3GalNAc (T-antigen) to PaTu-S and PaTu-T cells as measured by flow cytometry, and shown as average MFI ± SEM of at least 3 independent experiments. ^*^P ≤ 0.05 and ^***^P ≤ 0.001.

Since one of the most remarkable differences observed in GT expression between PaTu-S and PaTu-T was related to initiation of *O*-glycan synthesis, we studied this aspect in more detail. We determined for two selected mRNA species whether the differences in gene transcript levels between Patu-S and Patu-T were also observed at the protein level by Western blotting for the GT proteins. Substantial amounts of *GALNT3* and *CGNT3* protein levels were observed in whole cell lysates of PaTu-S, but were hardly detectable in PaTu-T, which correlated with the mRNA expression levels found in these cells (Figures 2, 3A). To define the potential of the cells to catalyze the addition of α-GalNAc to Ser/Thr residues on a peptide, a *ppGALNT* enzyme assay was performed using two different peptides with multiple Ser/Thr residues, derived from immunoglobin A (IgA) and mucin 2 (MUC2) proteins, respectively. PaTu-S cells showed a much higher *ppGALNT* activity that PaTu-T (Figure 3B), which was associated with elevated levels of surface Tn antigen as detected by using a monoclonal anti-Tn antibody (Figure 3C and Supplementary Figure S1A). Likewise, the activity of β1,3-galactosyltransferase (*C1GALT1*, T-synthase) converting Tn antigen into T antigen was measured and found to be highest in PaTu-S (Figure 3D). Human Jurkat T cells do not have T-synthase activity due to a mutation in the *C*osmc gene (*C1GALT1* chaperone) (27), and were therefore used as a negative and baseline control for the assay. In addition, the peanut agglutinin (PNA) showed enhanced binding to PaTu-S compared to the other cell lines, again indicating a relatively higher level of T-antigen (Figure 3E and Supplementary Figure S1B). In conclusion, PaTu-S cells show a higher potential for the synthesis of *O*-glycans, resulting in the presence of elevated levels of short *O*-glycans (both Tn and T antigens) compared to PaTu-T.

### *O*-Glycosylation Analysis in PaTu-S and PaTu-T by PGC Nano-LC-ESI-MS/MS

**Figure 4:**
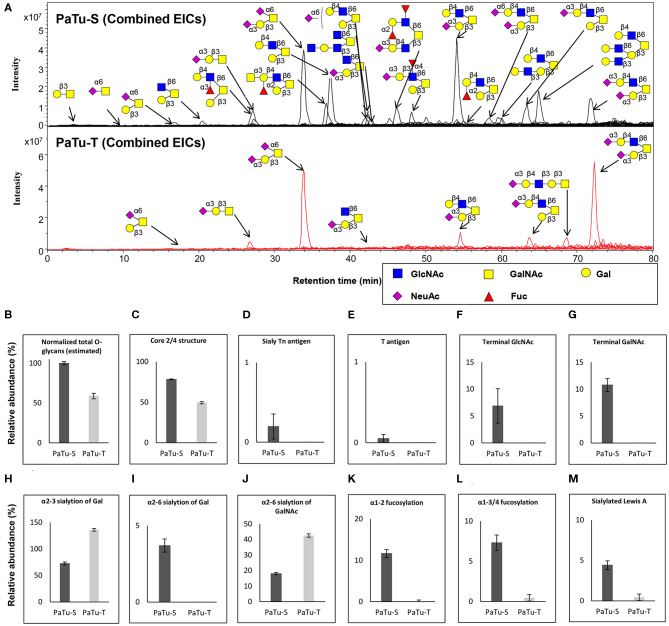
*O*-glycosylation analysis in PaTu-S and PaTu-T by PGC nano-LC-ESI-MS/MS. **(A)** Combined extracted ion chromatograms (EICs) of *O*-glycans derived from PaTu-S and PaTu-T cell lines. **(B–M)** Relative abundance of structural *O*-glycan classes derived from 0.5 million PaTu-S and PaTu-T cells on PGC nano-LC-ESI-MS/MS in negative ion mode (displayed as mean relative abundance plus standard deviation; *N* = 3). **(B)** Normalized total *O*-glycan content (estimated), **(C)** Core2/4 *O*-glycan structure, **(D)** Sialylated Tn antigen, **(E)** T antigen, **(F)** Terminal GlcNAc, **(G)** Terminal GalNAc, **(H)** α2,3-sialylation of Gal, **(I)** α2,6-sialylation of Gal, **(J)** α2,6-sialylation of GalNAc, **(K)** α1,2-fucosylation, **(L)** α1,3/4-fucosylation, and **(M)** sLe^A^.

To further study the total expression of cellular glycans, we analyzed the *O*-glycans released from PaTu-S and PaTu-T cells by PGC nano-LC-ESI-MS/MS (Figure 4A). PGC LC-ESI-MS/MS using negative electrospray ionization was employed for structural analysis of glycans, which is a powerful platform enabling discrimination between glycan isomers (28–30). Glycan structures were assigned on the basis of the obtained glycoanalytical information, and general glycobiological knowledge. The powerful chromatographic separation of two *O*-glycan isomers with *m/z* 675.30 and five GSL-glycan isomers with *m/z* 999.30 with characteristic MS/MS spectra are shown in Supplementary Figures S2, S3, respectively.

Representative *O*-glycan profile spectra with the main peaks annotated are shown in Figure 4A. Compared to PaTu-S, most of the *O*-glycans synthesized in PaTu-T, except for two disialylated *O*-glycans, were detected at lower level or depleted, resulting in less diversity of *O*-glycan species (Figure 4A) and a lower abundance of estimated total *O*-glycans (Figure 4B) in PaTu-T. Relative quantification of individual glycans is given in Supplementary Figure S4, revealing the large differences in detail. Glycan traits or ratios reflecting certain biosynthetic steps were calculated to facilitate the biological interpretation of the data and to relate the MS glycomics data to transcriptomics data. This included core 2/4 structures, sTn antigen, T antigen, terminal acetylglucosamine (GlcNAc), terminal *N*-acetylgalactosamine (GalNAc), α2,3 sialylation of galactose, α2,6 sialylation of galactose, α2,6 sialylation of GalNAc, α1,2 fucosylation of galactose, α1,3/4 fucosylation, and sLe^A^. In general, PaTu-T showed a lower levels of estimated total *O*-glycans, probably resulting from the lower level of *ppGALNTs* (Figure 4B). In PaTu-S, core 2 and core 4 *O*-glycans accounted for 78.2% of the species in contrast to only 49.4% in PaTu-T (Figure 4C) and in line with the higher expression of *GCNT3* in PaTu-S (Figure 2). Importantly, the tumor-associated *O*-glycans sTn antigen (Figure 4D) and T antigen (Figure 4E) were only detected in PaTu-S cell lines. Figures 4F,G further show that terminal GlcNAc and GalNAc were detected only in PaTu-S cells. For sialylation, an increase of α2,3 sialylation of galactose was observed for PaTu-S compared to PaTu-T (Figure 4H) in accordance with the elevated level of *ST3GALs* in PaTu-T cells (Figure 2). Interestingly, α2,6 sialylation on galactose was specifically present in PaTu-S cell line, as there was no α2,6-linked sialic acid on galactose in PaTu-T (Figure 4I). Opposite to α2,6 sialylation of galactose, sialylation on the GalNAc with α2,6 linkage was high in PaTu-T with a relative abundance at 42.5%, compared to 18.0% in PaTu-S (Figure 4J), which is in line with the expression patterns of *ST6GALNAC1, 4* and *6*. Notably, a decrease of α1,2 and α1,3/4 fucosylation was observed in the more mesenchymal-like PaTu-T cells (Figures 4K,L), in accordance with the decreased level of *FUTs* in PaTu-T cells (Figure 1). Remarkably, we also observed many specific glycan structures in PaTu-S cells including sLe^A^, blood H antigen, blood group A, and Lewis X (Figure 4A).

### Glycosphingolipid-Glycan Analysis in PaTu-S and PaTu-T by PGC Nano-LC-ESI-MS/MS

**Figure 5:**
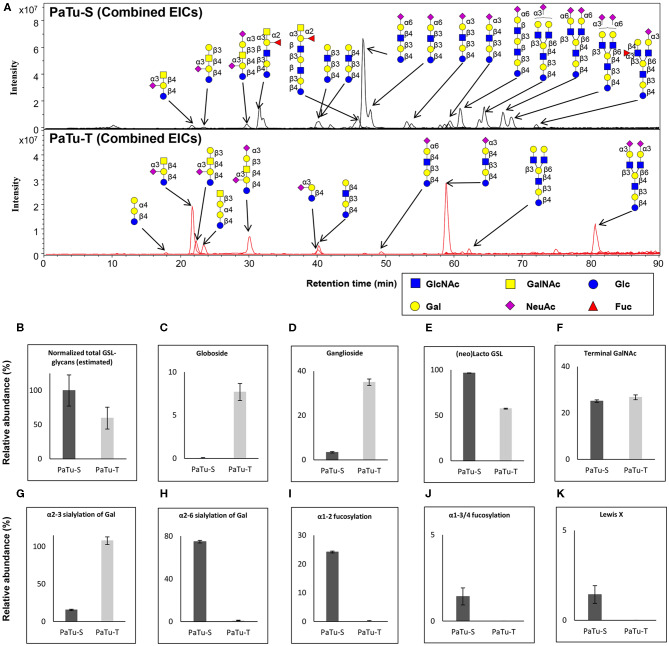
GSL-glycosylation analysis in PaTu-S and PaTu-T by PGC nano-LC-ESI-MS/MS. **(A)** Combined EICs of GSL-glycans derived from PaTu-S and PaTu-T cell lines. **(B–K)** Relative abundance of structural GSL-glycan classes derived from 2 million PaTu-S and PaTu-T cells on PGC nano-LC-ESI-MS/MS in negative ion mode (displayed as mean relative abundance plus standard deviation; *N* = 3). **(B)** Normalized total GSL-glycans content (estimated), **(C)** Globosides, **(D)** Ganglioside, **(E)** nsGSLs, **(F)** Terminal GalNAc, **(G)** α2,3-sialylation of Gal, **(H)** α2,6-sialylation of Gal, **(I)** α1,2-fucosylation, **(J)** α1,3/4-fucosylation, and **(K)** Lewis X.

Next, GSL-glycans were analyzed with PGC nano-LC-ESI-MS/MS after enzymatic release using endoglycoceramidase I (EGCase I) on purified GSLs derived from 2 × 10^6^ cells per sample. The combined extracted ion chromatograms of GSL-glycans in PaTu-S and PaTu-T cell lines (Figure 5A) and the relative quantification of GSL-glycans (Supplementary Figure S5) are presented. A lower abundance of estimated total GSL-glycans (Figure 5B) in PaTu-T was observed. The data showed vastly different GSL patterns of PaTu-T cells with higher levels of globosides and gangliosides and lower levels of nsGSLs (Figures 5C–E). Importantly, a specific detection of globosides (Gb3 and Gb4; 7.7±1.0%) in PaTu-T was found, indicating a possible important role of globosides in PDAC cells (Figure 5C). Approximately 10-fold higher levels of gangliosides were observed in PaTu-T cells (35.0 ± 1.4%) compared to PaTu-S cells (3.4 ± 0.3%). In contrast, nsGSLs were relatively lower in PaTu-T cells (57.4 ± 0.5%) compared to PaTu-S cells (96.6 ± 0.3%). These results are also in alignment with the gene expression data, which showed elevated expression of *A4GALT, ST3GAL5, B4GALNT1*, and a slightly decreased *B3GNT5* level (Figure 2).

Differences in GSL abundance between the two cell lines with respect to α2,3 and α2,6 sialylation of galactose residues, α1,2 fucosylation of galactose and α1,3/4 fucosylation were very much in line with those found for *O*-glycosylation (Figures 5G–J). Decreased abundance of α2,6 sialic acid on galactose (74.8 ± 1.2% in PaTu-S, Figure 5H), α1,2 fucosylation (24.2 ± 0.3% in PaTu-S, Figure 5I), and α1,3/4 fucosylation (1.4 ± 0.5% in PaTu-S, Figure 5J), were observed in PaTu-T compared to the PaTu-S. Remarkably, α2,3 sialylation of galactose was higher in the mesenchymal-like PaTu-T cells than in the epithelial-like PaTu-S cells (Figure 5G). No significant difference was found in terminal GalNAc of GSL-glycans between PaTu-S and PaTu-T (Figure 5F). Similar to *O*-glycans, the Lewis X moiety was found to be specifically detected on GSLs of PaTu-S cells (Figure 5K).

### Differential Abundance of Terminal Glycan Structures in PaTu-S and PaTu-T

In order to confirm the glycan expression and to understand the effects of the differential GT expression levels described above, the binding of different glycan-binding proteins (GBPs) including lectins and anti-glycan antibodies to the cell lines was analyzed by flow cytometry.

**Figure 6:**
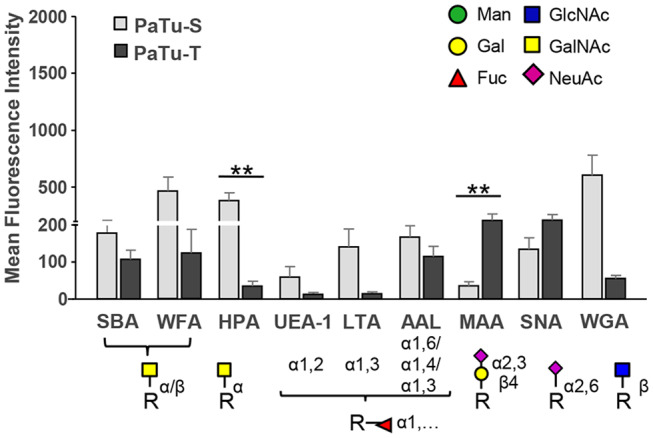
Binding of glycan-binding proteins to PaTu-S and PaTu-T cells. Lectin binding to PaTu-S and PaTu-T cells was measured by flow cytometry. The schematic glycan structures which are shown under the lectins used are common structures known to be recognized by the lectins. Results are shown as average MFI ± SEM. ^**^P ≤ 0.01.

As shown in Figure 6 and Supplementary Figure S6, the lectins *Helix pomatia* agglutinin (HPA; terminal α GalNAc), soybean agglutinin (SBA; terminal α/βGalNAc and Gal) and *Wistera floribunda* (WFA; terminal α/βGalNAc) showed enhanced binding to PaTu-S compared to PaTu-T. Together with the observation that several *GALNT* genes were expressed at higher levels in PaTu-S compared to PaTu-T, these data indicate the presence of relatively higher amounts of terminal GalNAc residues on PaTu-S. In addition, PaTu-S showed substantial binding of the Wheat germ agglutinin (WGA; βGlcNAc), suggesting the presence of structures with terminal GlcNAc. These results are in accordance with the higher abundance of terminal GlcNAc in *O*-glycans (Figure 4F).

By contrast, PaTu-T cells showed higher binding of the α2,3 sialic acid-recognizing lectin *Maackia amurensis* (MAA, α2,3 sialic acids) than PaTu-S, which was in agreement with a higher expression of *ST3GAL3, -4* and *-6* genes and a higher abundance of α2,3 sialylation of galactose in both *O*-glycans (Figure 4H), GSL-glycans (Figure 5G), and *N*-glycans (24) in PaTu-T (Figure 5G). The binding of SNA lectin, specific to α2,6 sialic acids, to PaTu-T cell lines was elevated, indicating higher levels of α2,6 sialylation in PaTu-T. More details on the relevance of transcriptomics and MS glycomics data will be discussed in the discussion.

*Lotus tetragonolobus* lectin (LTA) bound almost exclusively to PaTu-S, indicating the presence of terminal α1,3/4-linked fucose. In addition, binding of UEA-1, which detects terminal α1,2-linked fucose (H-antigen), to the surface of PaTu-S cells was observed, which is in agreement with the higher levels of *FUT1/FUT2* transcripts (25) and the higher fucosylation level in *O*-glycans (Figures 4K,L) and GSL-glycans in these cells (Figures 5I,J). In addition, considerably higher levels of sLe^A^ structures were found in *O*-glycans of PaTu-S than those of PaTu-T (Figure 4M), indicating the presence of sLe^A^ antigen on PaTu-S, in line with our previous findings of more anti-sLe^A^ antibody binding to PaTu-S than to PaTu-T (24).

### PaTu-S and PaTu-T Differentially Bind to Lectins of the Immune System

**Figure 7:**
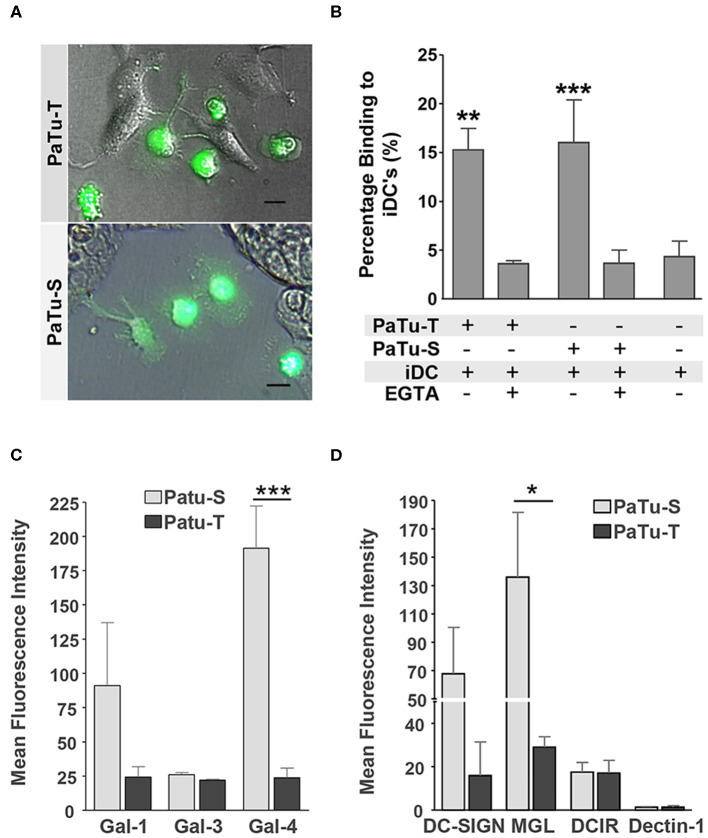
Immune recognition of glycan structures on PaTu-S and PaTu-T cells. **(A)** Interaction of immature DCs with PaTu-S and PaTu-T were visualized by fluorescence microscopy. Bar = 100 μm. **(B)** Binding of immature DCs to PaTu-S and PaTu-T in a cell adhesion assay, in the presence or absence of EGTA. Results are derived from 6 independent experiments using different donors and expressed as average percentage binding ± SEM. **(C)** Binding of recombinant human galectins Gal-1, Gal-3, and Gal-4 (5 μg/ml) to the PDAC cell lines was measured by flow cytometry. Results are given as average MFI ± SEM of at least 2 independent experiments. **(D)** Binding of Fc-chimeras of DC-SIGN, MGL, DCIR and Dectin-1 to PaTu-S and PaTu-T cells was measured by flow cytometry. Results are given as average MFI ± SEM of at least 3 independent experiments. ^*^P ≤ 0.05, ^**^P ≤ 0.01, and ^***^P ≤ 0.001.

In the immune system, dendritic cells (DCs) are specialized in the control of innate and adaptive immunity by sensing of pathogens as well as cancer cells via receptors present on their cell surface (31). The different glycan structures on PaTu-S and PaTu-T will very likely provoke a differential interaction with C-type lectin receptors present on DCs lectins, as well as with soluble immunolectins like galectins. Such an interaction could directly influence immune responses toward the tumor cells, or mediate immune escape. Here, the interaction of immature DCs with both PDAC cell lines was observed by fluorescence microscopy (Figure 7A) and the level of binding was quantified using a cell adhesion assay (Figure 7B). The DCs showed a similar level of binding to the two PDAC cell lines. This binding was C-type lectin dependent, since it was abrogated in the presence of the chelating agent EGTA (Figure 7B). In addition, the binding of recombinant soluble galectins (Gal-1, Gal-3, and Gal-4) and lectins (DC-SIGN, MGL, DCIR, and Dectin-1), which are normally present on the surface of immature DCs, to both PaTu cell lines was studied. The results showed that whereas the dendritic cell immunoreceptor (DCIR) bound both cell lines, and no binding of Dectin-1 could be detected. Macrophage galactose-type lectin (MGL) and dendritic cell-specific intercellular adhesion molecule-3-grabbing non-integrin (DC-SIGN) showed a differential binding pattern, with elevated binding to PaTu-S cells, compared to PaTu-T (Figure 7C). In addition, more Gal-1 and Gal-4 bound to PaTu-S compared to PaTu-T, whereas there was no difference in binding of Gal-3 to both cell lines observed (Figure 7D). We have previously shown that the binding of Gal-4 to both cell lines was glycan dependent (22). Although the binding of both Gal-1 and Gal-3 has not been tested for exclusively be glycan-dependent, it is assumed to be glycan-dependent, without excluding non-glycan dependent interactions. Because there is hardly any binding by Gal-3 detected on both cell lines, this indicates binding specific by Gal-1. These data indicates differences in lectin binding properties between PaTu-S and PaTu-T suggest a disparate effect on the immune response, the exact nature of which is subject for future investigations.

## Discussion

Alterations in glycosylation of cancer cells play an important role in the progression of cancer, however the individual roles that specific glycans play are incompletely understood. A rational design of experimental approaches to selectively detect and functionally analyze the role of specific glycans in the progression of PDAC requires a detailed understanding of the biosynthetic pathways that lead to cancer-associated glycan expression. Recently a direct correlation was reported between the formation of CA 19-9 and the development of rapid and severe pancreatitis leading to pancreatic cancer in an inducible CA 19-9 expression mouse model (3). In this model the elevation of CA19-9 observed in PDAC patients was mimicked. Engle et al. showed that the expression of *FUT3* and *B3GALT5* in mouse PDAC cells were essential to the cell surface expression of CA 19-9. In addition, the authors showed that the expression of glycan CA 19-9 together with Kras^G12D^ oncogene was a causal origin of aggressive development in pancreatic tumorogenesis. The work from Engle et al. show that the alteration in expression of specific glycosyltransferases and therefore their glycan products could be pivotal in tumor development.

Tumor metastasis formation and development evolves in a multistep process: (1) local invasion, where tumor cells undergo epithelial to mesenchymal transition (EMT) leading to (2) intravasation, where cells penetrate into the blood or lymphatic system; (3) extravasation, where cells from the circulation extravasate into the tissues of distant organs; (4) and finally colonization where cells undergo mesenchymal to epithelial transition (MET)-driven cell proliferation leading to the formation of secondary tumors (32–34). In order to increase insight in these processes, we studied the differential glycosylation of the mesenchymal-like PaTu-T and the epithelial-like PaTu-S cell lines. These PDAC cells show a high and low metastasizing capacity, respectively, in a *Danio rerio* model (21, 22), however in a mouse model (20) these cells show opposite outcomes. Both models are validated (21, 35) and the difference in the set-up of these models may explain these opposite outcomes. Both cells lines originated from a secondary tumor of a human patient, indicating that both cells had undergone all tumor developmental and metastasis stages. The mouse model applied by Elsasser et al. (20), where cells were injected intravenously into the tail vein of nude mice, may be considered as a tumor cell extravasation model since the cells were directly injected into the blood stream. This mouse model may be not fully physiological, since the cells often end up in the microvasculature of the lungs, resulting in local tumor growth (33) and thus focus more on the survival and colonization of PaTu-S cells exclusively in the lungs (20). By contrast, in the zebrafish model, the cells have been injected in the yolk-sac, and PaTu-T but not PaTu-S cells had the capacity to migrate into the blood circulation. In addition, PaTu-T cells have been shown to induce the first stages of metastasis including the formation of micro-metastases (21, 22). Nevertheless, further development into macro-metastasis was not possible due to the inherent short time constrain of the model. Therefore, both models provide important information about the behavior of these cells in tumor development and metastasis depending of the setting they are studied, and thus represent valuable model systems for functional studies regarding PDAC progression.

Using a comprehensive glycomics study, we showed major differences in the expression levels of glycan-related genes, in particular genes encoding GTs, between PaTu-S and PaTu-T. In previous work, we showed that the gene expression levels of enzymes involved in core *N*-glycosylation do not demonstrate remarkable differences between PaTu-S and PaTu-T, and that differences mainly occurred at *N*-glycan termini (24). By contrast, in this study we demonstrate significant changes in the expression of genes involved in *O*-glycosylation and GSL-glycosylation, some of which were confirmed to be reflected at the glycan level by Western blotting, binding to plan lectins and interaction with immunolectins. Combined, these results suggest a more dominant role of *O*-glycosylation and GSL-glycosylation in pancreatic cancer biology, compared to *N*-glycosylation.

One of the most remarkable differences between the two cell lines is observed at gene transcript levels of enzymes involved in synthesis of core 1/2 *O*-glycans, which are important determinants of the mucin-layer of epithelial cells. Mucins, especially the highly-glycosylated MUC1, MUC4, MUC5AC, and MUC16, are prominently observed in pancreatic cancer and major carriers of glycans including the CA 19-9 antigen (36, 37). Mucin-type *O*-glycans usually undergo drastic changes during PDAC progression (13, 18, 36), thereby influencing important properties of the tumor cells, including growth, adhesion, and interactions with the immune system. The enhanced mucin-type *O*-glycan synthases (*ppGALNTs)* and T-synthase activity, as well as enhanced levels of the Tn antigen and T antigen observed in PaTu-S compared to PaTu-T, demonstrate an elevated biosynthesis of short mucin-type *O*-glycans in PaTu-S. The expression of both antigens is mainly regulated by *ppGALNTs* and inefficient conversion of Tn antigen to T antigen. The inefficient conversion to T antigen is regulated by activity of T synthase and Cosmc, which is a chaperone involved in the proper folding and activity of T-synthase (38). Such inefficient Cosmc expression could be due to lowered expression caused for example by epigenetic silencing (17, 39). In addition, knocking out of *C1GALT1* in PDAC cells leads to inefficient conversion of Tn antigen to T antigen, and further increases tumor progression and metastasis (40). In these cells, the presence of Tn antigens most probably is due to a strongly enhanced expression of *ppGALNT* genes, encoding a family of enzymes that catalyze the first synthetic step in mucin-type *O*-glycosylation, including *GALNT3*, -*T4, -T5, -T7*, and -*T12*. This may result in an extended *O*-glycan initiation platform in PaTu-S cells as compared to PaTu-T. Consequently the higher expression of these *ppGALNTs* could possibly lead to an incomplete conversion of Tn to T antigen by active T-synthase or a relocation of the GalNAc-Ts from the Golgi apparatus to the endoplasmic reticulum (10). Similar to PaTu-S, a high *GALNT3* expression was observed in human pancreatic cancer tissues (13). Suppression of its expression significantly decreased cell growth and increased apoptosis (13). Recently, loss of *GALNT3* in poorly differentiated PDAC was shown to alter glycosylation of Erythroblastic Leukemia Viral Oncogene Homolog (ErbB) family proteins, and further associated with increased tumor aggressiveness (19). *GALNT3* expression was found to be restricted to glandular epithelial cells (41) in lung adenocarcinoma and to be downregulated in mesenchymal like cells in epithelial-mesenchymal transition models (42, 43). *GALNT7* upregulation has previously been shown to be involved in growth and invasion in cervical cancer (44) and its downregulation increases metastasis formation in melanoma cells (45). These findings agree with our data, showing a much higher expression of *GALNT3* and *GALNT7* in the more differentiated epithelial-like PaTu-S, compared to the mesenchymal-like PaTu-T cells. In addition to an elevated level of Tn antigen, also elevated levels of T antigen were found in PaTu-S compared to PaTu-T cells. This indicates that despite the high expression of *GCNT3* in PaTu-S cells, the T antigens were not efficiently converted to core 2 structures. In contrast to PaTu-S, the mesenchymal-like PaTu-T expressed lower levels of Tn synthases, which could explain the decrease in total *O*-glycan expression. The expression of certain mucins was elevated in PaTu-S, however, PaTu-T showed increased expression of other glycoproteins such as mucolipins (data not shown). Therefore, it cannot be excluded that lower expression of glycoproteins with many *O*-glycan attachment sites such as mucins contributes to the effect. In addition, elevated *ST6GALNAC4* and *6* terminate β1-6 branching of core GalNAc, prohibiting the conversion to core 2/4 structures, which may result in a lower abundance of core 2/4 in PaTu-T.

GSLs play essential roles in maintenance of membrane stability and regulation of numerous cellular processes, including adhesion and metastasis (46–48). Our result shows higher level of *UGCG, B4GALT5*, and *B4GALT6* in the mesenchymal-like PaTu-T compared to PaTu-S. Notably, neoexpression of globosides (Gb3 and Gb4) was observed, matching the increased expression level of *A4GALT*. This is an interesting observation, since it has been reported that both *UGCG* and Gb3 are associated with tumor metastasis. Inhibition of *UGCG* reduced the levels of all cellular GSLs, including lactosylceramide, Gb3 and GM3, thus decreasing lung tumor metastasis (49). Moreover, enhanced ceramide glycosylation by *UGCG* has been shown to confer multidrug resistance of cancer cells (50) and to selectively maintain pluripotency properties of cancer stem cells (51). Globoside Gb3 has also been reported to be highly expressed in a number of tumors, including metastatic colon cancer (52, 53), breast cancer (54), gastric adenocarcinomas (55), and pancreatic cancer (56). Importantly, the overexpression of Gb3 in cultured human colonic epithelial cells induced cell invasiveness, whereas knocking Gb3 down inhibited cell invasiveness (52). We show in this study that globoside Gb3 and Gb4 are specifically expressed in the mesenchymal-like PaTu-T cells, indicating it may contribute to the metastasis in PDAC. However, further functional studies are necessary to further understand the role of globosides especially Gb3 in cancer progression and metastasis.

A striking difference in terminal glycosylation was observed in the levels of fucosylated structures. Fucose, as a constituent of oligosaccharides, is often associated with cancer and inflammation (57, 58), and can be used as a promising target for cancer diagnosis and therapy (59). The enhanced binding of UEA1 to PaTu-S compared to PaTu-T is in line with the higher levels in PaTu-S of the FUT genes *FUT1* and *FUT2*, which are involved in the synthesis of the H-antigen, the precursor for blood group A/B antigens (BG-A/B). This can be further confirmed by highly expressed blood group H antigen and BG-A in PaTu-S compared to PaTu-T (Supplementary Figures S7A–C). These data are consistent with our previous report (25) where we showed that both the expression of *FUT1* and *FUT2* genes, together with the binding of anti-BGA and anti-BGB antibodies, using flow cytometry, were much higher in PaTu-S and undetectable in PaTu-T cells. In addition, the α1,3 FUT gene *FUT4* is expressed at higher levels in PaTu-S and may be involved in the synthesis of Lewis X (24), and in combination with *FUT1* and *FUT2* in PaTu-S in the synthesis of Lewis Y. The observation that higher binding of the lectin LTA, which binds to terminal α1,3 fucose, to PaTu-S than to PaTu-T as well as the specific presence of Lewis X on *O*-glycans (Supplementary Figure S7D) and GSL-glycans in PaTu-S is also in agreement with our previous reports (24, 25) and supports the expression of these known cancer-associated glycan structures (14) on PaTu-S cells.

Altered sialylation has long been associated with metastatic behavior of tumor cells including invasion and enhanced cell survival [reviewed in (60)]. Overexpression of *ST3GAL3* in pancreatic cell lines induced increased expression of sLe^X^ together with decreased α2,6 sialylation on α2β1 integrin and E-cadherin, which further regulate adhesion and invasion in pancreatic cancer (61–63). The mesenchymal-like PaTu-T shows enhanced binding of the α2,3-sialic acid-recognizing lectin MAA compared to the epithelial-like PaTu-S, which is in line with the higher expression levels of *ST3GALs* in PaTu-T for all these three types of glycans. For the α2,6-linked sialic acid, we observed higher SNA lectin binding to PaTu-T cells indicating a slightly higher expression level of α2,6 sialic acid. However, we found a reduced relative abundance of α2,6 sialylation of galactose in all three types of glycans [*O*-, GSL-, or *N*-glycans (24)] together with decreased expression of *ST6GAL1* in PaTu-T when compared to PaTu-S. This conflicting result could be explained by the higher expression level of α2,6-linked sialic acid on GalNAc in *O*-glycans (Supplementary Table S1). This type of α2,6 sialylation of GalNAc is highly expressed in the mesenchymal-like PaTu-T cells, in accordance with the increased gene expression of *ST6GALNAC 4* and *6*. Similar to PaTu-T, *ST6GALNAC4* was shown to be upregulated in other mesenchymal type PDAC cells (42).

The profound differences in glycosylation between PaTu-S and PaTu-T may indicate that these cells will differentially interact with immune cells. The preferential binding of DC-SIGN to PaTu-S may be due to the higher abundance of fucose-containing Lewis antigens (64), whereas the higher MGL binding to PaTu-S can be explained by the higher levels of terminal GalNAc, including the Tn antigen. Binding of DCs to cancer cells via MGL and DC-SIGN was previously shown and has been proposed to lead to an immune escape of the tumor cells (65–67). Since MGL binds to terminal α- and β-linked GalNAc residues, including Tn-antigen (65, 68), the differential binding of MGL and DC-SIGN to the cell lines corresponds well with their respective glycosylation profiles, especially in *O*-glycans. Expression of the Tn antigen on cancer cells is associated with poor prognosis in several types of cancer and instructs DCs to drive Th2 mediated responses, which contribute to tumor development (31).

Also the galectins 1 and 4 show differential binding to PaTu-T and PaTu-S. Galectins belong to a family of soluble immunomodulatory proteins that may act extracellularly by crosslinking their ligands thereby modulating signaling pathways (31). Remarkably, the soluble lectin Gal-4 shows a much higher binding to PaTu-S than to PaTu-T (8-fold), which may be due to the presence of blood group H and A ligands for this lectin on PaTu-S (25, 69). We demonstrated previously that PaTu-S, but not PaTu-T produces significant levels of intracellular and secreted Gal-4 (22). Whereas cytoplasmic Gal-4 can suppress metastatic activity in PDAC cells (22, 23), the role of extracellular Gal-4 in PDAC is unknown, but may include adhesion of the tumor cells to each other, or to extracellular matrix structures.

## Conclusion

In this work an overview of the difference in cellular glycosylation between two cell lines was obtained using a variety of approaches. Supplementary Table S1 provides an overview on the significant alterations identified in this work and our previous studies (24, 25). The observed differences in expression levels of GTs are in good accordance with the glycan structures identified by mass spectrometry and flow cytometry. This is remarkable, since cancer cells are often reported to display a disorganized ER/Golgi compartment, which would be expected to influence glycosylation patterns (10). Moreover, by using this combined approach it was possible to demonstrate the association of specific glycan structures with their carrying glycan types (Supplementary Table S1). For example, we showed detailed changes in α2,6 sialylation as well as that Lewis X is predominantly detected on GSLs and *O*-glycans while sLe^A^ was mainly found on *O*-glycans. In addition, the higher binding ability of HPA, SBA, and WFA lectins to terminal GalNAc in PaTu-S was in accordance with the increased level of terminal GalNAc in *O*-glycosylation, rather than present on GSL-glycans, highlighting the importance of studying cellular glycosylation in a comprehensive and glycan-specific manner for characterization and functional studies of glycosylation.

The epithelial-like PaTu-S cells and the more mesenchymal PaTu-T cells can be regarded as a differentiation model of cells that undergo the early and later stages of metastasis formation. Here we show pronounced differences in *O*- and GSL-glycosylation between PaTu-T and PaTu-S cells, which is in line with the observed gene expression and associated with differential interaction with immune cell lectins. The results from this study provide leads for future research to define the role of specific glycan structures in PDAC progression, such as the role of the globoside Gb3 in metastasis, which may reveal targets for future prognostic and/or therapeutic approaches. Recent work has opened new possibilities for pancreatic cancer prevention and possible pancreatitis treatment through direct targeting of CA 19-9 (3). Aberrant glycosylation leads changes in key factors that lead to cancer development, poor prognosis, and treatment resistance (70). The possibilities and the necessity of current approaches in personalized cancer therapy by tackling these changes in glycosylation are emerging and becoming more prominent.

## Materials and Methods

### Materials, Chemicals, Antibodies, Reagents, and Buffers

Ammonium bicarbonate, cation exchange resin beads (AG50W-X8), trifluoroacetic acid (TFA), potassium hydroxide, ammonium bicarbonate, and sodium borohydride were obtained from Sigma-Aldrich (Steinheim, Germany). 8 M guanidine hydrochloride (GuHCl) was obtained from Thermo Fisher Scientific. Peptide N-glycosidase F (PNGase F) was purchased from Roche Diagnostics (Mannheim, Germany). Endoglycoceramidase I (EGCase I, recombinant clone derived from Rhodococcus triatomea and expressed in *Escherichia coli*), 10x EGCase I buffer (500 mM HEPES, 1M NaCl, 20 mM DTT and 0.1% Brij 35, pH 5.2), glycobuffer 1 (50 mM sodium acetate, 5mM CaCl_2_, pH 5.5), and α2–3 neuraminidase S were purchased from New England BioLabs Inc., (Ipswich, MA, USA). Dithiothreitol (DTT), HPLC SupraGradient acetonitrile (ACN) was obtained from Biosolve (Valkenswaard, The Netherlands) and other reagents and solvents such as chloroform, methanol, ethanol, 2-propanol, and glacial acetic acid were from Merck (Darmstadt, Germany). MultiScreen^®^ HTS 96 multiwell plates (pore size 0.45 μm) with high protein-binding membrane (hydrophobic Immobilon-P PVDF membrane) were purchased from Millipore (Amsterdam, The Netherlands), conical 96-well Nunc plates from Thermo Scientific (Roskilde, Denmark). The 50 mg TC18-reverse phase (RP)-cartridges were from Waters (Breda, The Netherlands). Ultrapure water was used for the all preparations and washes, generated from a Q-Gard 2 system (Millipore, Amsterdam, The Netherlands).

Rabbit anti-human *GALNT3* and anti-human *GCNT3* (Atlas Antibodies, Stockholm, Sweden) antibodies (Abs) were used for detection of *GALNT3* and *GCNT3*, respectively. Mouse anti-human galectin (Gal)-1 (71), and anti-Tn monoclonal antibodies were kindly provided by Dr. RD Cummings (Boston, USA), goat anti-human Gal-4 was purchased from R&D Systems (Minneapolis, MN) and anti-sialyl Lewis A/CA 19-9 monoclonal antibody was from LifeSpan Biosciences (Seattle, WA). Goat anti-human actin (Santa Cruz Biotechnology, Heidelberg, Germany) was used as an endogenous control. Secondary Abs and proteins used were goat anti-rabbit Alexa Fluor 488 (AF 488), anti-mouse AF 488 and rabbit anti-goat AF 488, streptavidin AF 488 (Molecular Probes, Invitrogen, Carlsbad, CA), goat anti-human Fc (Jackson Immunoresearch, Suffolk, UK) and IRDye 680 anti-goat and IRDye 800CW anti-rabbit IgG (LI-COR Biosciences, Lincoln, Nebraska). Roti-Block blocking buffer was acquired from Roth (Karlsruhe, Germany) and Calceine AM from Molecular Probes. Recombinant human Gal-1 (72) was kindly provided by Dr. Rabinovich. Production of biotinylated recombinant human Gal-3 was described in van Stijn et al. (73) and recombinant human Gal-4 was purchased from R&D. Recombinant C-type lectins [MGL-Fc (74), DC-SIGN-Fc (75), DCIR-Fc (76), and Dectin1-Fc] consist of the extracellular domains of the lectins fused to the human immunoglobulin IgG1-Fc tail. To construct Dectin1-Fc, the extracellular domains of human Dectin-1(a) (amino acids 65-241) were amplified using total RNA from immature DCs and fused at the C-terminus to human IgG1-Fc in the Sig-pIgG1-Fc vector. Dectin-1-Fc was produced by transient transfection of HEK-293T cells.

Biotinylated lectins from peanut (PNA) and *Helix pomatia* (HPA) were purchased from Sigma-Aldrich (Zwijndrecht, The Netherlands). Lectins from *Aleuria aurantia* (AAL), *Lotus tetragonolobus* (LTA), *Maackia amurensis* (MAA; mixed MAA-I and MAA-II), soybean agglutinin (SBA), *Sambucus nigra* agglutinin (SNA), *Ulex europaeus I* (UEA-1), *Wistera floribunda* (WFA), and Wheat germ agglutinin (WGA) were bought from E-Y Laboratories (San Mateo, CA).

### Cells and Cell Culture

PaTu-S and PaTu-T cell lines were purchased at DSMZ culture bank (Braunschweig, Germany). Cell lines were cultured as previously described Belo et al. (22). Cell lines were monthly tested to verify the absence of mycoplasma, and human cell lines were authenticated by short tandem repeat (STR) analysis. Human monocytes isolated from healthy donor buffy coats (Sanquin Blood Bank, Amsterdam, The Netherlands) were differentiated to immature dendritic cells as described van Stijn et al. (73).

### Microarray Analysis

Cells were harvested at 60% confluency using trypsin-EDTA (Gibco, Invitrogen). mRNA was isolated using the RNeasy Mini kit (Qiagen, Venlo, The Netherlands), following the manufacturers protocol. The quality of the mRNA was established by measurement of the A260/A280 ratio and in-gel agarose visualization. Microarray screening was performed by the Consortium for Functional Glycomics (CFG) (http://www.functionalglycomics.org), using the GlycoV4 oligonucleotide array, a custom Affymetrix GeneChip (Affymetrix, CA, USA) designed for the CFG. This array includes ~1,260 human probe-ids related to glyco-genes. The data were normalized using RMA Express 1.0 (http://rmaexpress.bmbolstad.com) with quantile normalization, median polish, and background adjustment. Differential expression was calculated using the Limma package in R software. Fold change and standard errors were estimated by fitting a linear model for each gene and empirical Bayes smoothing was applied to the standard errors. The adjusted *p*-value is the *p*-value adjusted for multiple testing using the Benjamini and Hochberg's method (77) to control the false discovery rate of 0.1 or less. The transcripts identified as differentially expressed were those with adjusted *p* < 0.1 and a difference in fold change > 1.5. Cluster Analysis and TreeView Visualization Software was used to visualize the microarray data. Both programs are integrated for analysis and visualization of complex microarray experiments data. Both software programs were developed by Michael Eisen at the Michael Eisen's lab in the Howard Hughes Medical Institute (HHMI) at University of California at Berkeley (UCB) and the Lawrence Berkeley National Lab (LBNL) USA.

### Quantitative Real-Time PCR

mRNA was isolated from cells at 60% confluence using the mRNA Capture kit (Roche, Basel, Switzerland) according to the manufacturer's guidelines. cDNA was synthesized with Reverse Transcription System kit (Promega, San Luis Obispo, CA). Part of the oligonucleotides used were previously described Garcia-Vallejo et al. (78). The remaining oligonucleotides used in this study were designed using software from http://www.ncbi.nlm.nih.gov/nuccore, and were synthesized by Invitrogen (Table 1). PCR reactions were performed as described Belo et al. (22) and relative mRNA abundance was calculated from CT values from the target vs. the endogenous reference gene glyceraldehyde-3-phosphate dehydrogenase (*GAPDH*) (78).

### Western-Blotting

Shortly, 150 μg total proteins of whole cell lysates were separated by SDS-PAGE and the proteins were transferred to nitrocellulose membranes (Whatman Protran, Sigma). Blots were incubated with anti-*GALNT3* and *GCNT3* Abs (0.1 μg/ml) and anti-Actin Abs (0.2 μg/ml, as a loading control), followed by incubations with the secondary IRDye Abs (0.07 μg/ml). Western-blot analysis was performed using LI-COR Odyssey systems scanner and software.

### Glycosyltransferase Activity

Cells were lysed in TSM buffer containing protease inhibitors by five sonication cycles of three sec each on ice after which Triton X-100 (0.5% final concentration) was added and cell lysates were further incubated on ice for 20 min. Lysates were centrifuged at 1000 × *g* at 4°C for 5 min and supernatant was used in both T-synthase and GALNT assays. T-synthase activity was measured as described in Ju et al. (79), using GalNAc-α-(4-MU) (1,000 μM) as an acceptor, UDP-Gal (500 μM) as a donor, and the cell extracts as enzyme source. Cell extracts from human Jurkat T cells were used as negative control. For the standard curve measurements, free 4-MU was used. Reaction mixtures were incubated for 60 min at 37°C and the final fluorescent product was measured on a FLUOstar Spectrofluorimeter (excitation 360 nm and emission at 460 nm).

GALNT assays were carried out in a volume of 50 μl reaction mixture (pH = 7.0), containing 0.1 M sodium cacodylate, 10 mM MnCl_2_, 4 mM ATP (to counteract enzymatic degradation of the nucleotide sugar), 0.5% Triton X-100, 0.25 mM UDP-[^3^H]-GalNAc (4,15 Ci/mol), 1 mM acceptor peptide [PVPSTPPTPSPSTPPTPSPS, comprising the IgA hinge region (80)] and PTTTPITTTTTVTPTPTPTGTQT, representing MUC2 tandem repeat region, and 5 μl cell lysate as enzyme source. After a 4 h incubation at 37°C, the radioactive products were isolated using Sep-Pak C-18 cartridges (Waters, Milford, MA) as described Palcic et al. (81). Incorporation of radioactivity in the peptides was assessed using a liquid scintillation MicroBeta2 Plate Counter 2450 (Perkin Elmer, USA). Control assays lacking the acceptor were carried out to correct for incorporation into endogenous acceptors.

### *O*-Glycan Release and Purification From PaTu-S and PaTu-T Cell Lines

We have previously described a high-throughput polyvinylidene difluoride (PVDF)-membrane-based 96-well plate for *N*-glycans release (24). Here, a sequential *N*-glycan release followed by *O*-glycan release by β-elimination was established to achieve reduced *O*-glycans derived from each cell line. After desalting and purification, the *O*-glycans were subjected to analysis on PGC nano-LC-ESI-MS/MS (82). Cell pellets with ~2 × 10^6^ cells from three biological replicates of PaTu-S and PaTu-T were washed with PBS and suspended in 100 μl H_2_O. A high-throughput release of *N*-glycans has been performed first to avoid contamination during *O*-glycan release as previously described Holst et al. (24). After removal of *N*-glycans, the *O*-glycan release via β-elimination, desalting and PGC clean-up was carried out in a high-throughput method using a PVDF membrane-based protocol adapted from Jensen et al. (82). Briefly, 60 μl of 0.5M NaBH4 in 50 mM KOH were applied onto the PVDF membrane in each well of 96-well plates after rewetting the PVDF membrane by 3 μl of methanol. Plates were shaken for 30 min on a horizontal shaker and incubated in a moisturized, sealed box as an incubation chamber within an oven for 16 h at 50°C. After incubation and cooling to RT, released *O*-glycans were recovered by centrifuge at 1,000 × g for 2 min into 96-well collection plates. The wells were rewet by 3 μl of methanol and washed by 50 μl of H_2_O with 10 min incubation steps on a horizontal shaker prior to centrifugation at 1,000 × g for 2 min. This wash and centrifugation steps were performed three times, and the washes were pooled to the collection plates.

Prior to desalting of *O*-glycans, the collected samples were concentrated to ~30 μl under vacuum in an Eppendorf Concentrator 5301 (Eppendorf, Hamburg, Germany) at 30°C for 2 h. Subsequently, 3 μl of glacial acetic acid was added to neutralize the reaction followed by a brief centrifugation to collect the sample at the bottom of the well. In the next step, desalting of *O*-glycans was performed on cation exchange solid phase extraction (SPE) which consisted of 80 μl of AG50W-X8 resin slurry deposited onto OroChem 96-well filter plates (Perfect Pure, Millipore) as previously described Jensen et al. (82). Glycan samples were loaded onto the plates, and flow-through was collected into a collection plate after centrifugation at 1,000 × g for 2 min. Glycan alditols were eluted with 50 μl of water twice. The combined flow-through and eluate were pooled and dried under vacuum in an Eppendorf Concentrator at 30°C. Subsequent drying steps with 100 μl of methanol were performed thrice to remove residual borate. As the last step, the carbon SPE clean-up of *O*-glycans was performed following the same procedure with adjustment to a 96-well filter plates (82). Briefly, 80 μl of PGC slurry was loaded onto OroChem 96-well filter plates, and a two-step preconditioning were performed with 100 μl of 80% ACN in H_2_O with 0.01% TFA followed by 100 μl of H_2_O with 0.01% TFA. The columns were washed 3 × 50 μl of H_2_O with 0.01% TFA after loading samples. *O*-glycans were subsequently eluted by 40 μl of 60% ACN in H_2_O with 0.01% TFA and dried in concentrator. The purified glycan alditols were re-suspended in 10 μl of water prior to PGC nano-LC-ESI-MS/MS analysis.

### Glycosphingolipid Extraction and Purification by RP-SPE

Extraction of GSLs from PaTu-S and PaTu-T cell lines was performed in triplicate using glass centrifuge tubes. Cells were harvested (2 × 10^6^) and washed three times in 1 ml of water followed by centrifugation at 2,000 × g for 30 min. The supernatant was removed and replaced by 200 μl of water. The cell samples were vortexed for 5 min and sonicated (Ultrasonic Cleaner, VWR) for 30 min. Chloroform (550 μl) was added to the samples followed by 15 min sonication. Methanol (350 μl) was added to the cell pellets and incubated for 4 h with shaking at room temperature. The upper phase containing GSLs was collected after centrifugation at 2,700 × g for 20 min. Then, 400 μl of chloroform/methanol (2:1, v/v) was added, followed by adding 400 μl of methanol/water (1:1, v/v). After sonication and centrifugation, the upper phase was collected and pooled to the previous sample. The process of adding methanol/water (1:1, v/v), sonication, centrifugation and removing upper phase was repeated for another two times. In each replicate, the upper phase was collected and replaced by the same volume of methanol/water (1:1, v/v). The combined upper phases were dried under vacuum in an Eppendorf Concentrator 5301 (Eppendorf, Hamburg, Germany) at 30°C.

Before the purification of the GSLs using reverse phase (RP) SPE, the samples were dissolved in 100 μl methanol and vortexed for 10 min, followed by the addition of 100 μl water. TC18-RP-catridges were prewashed with 2 ml of chloroform/methanol (2:1, v/v), 2 ml of methanol followed by equilibration with 2 ml methanol/water (1:1, v/v). The extracted GSLs were loaded to the cartridge 3 times and washed with 2 ml methanol/water (1:1, v/v). The GSLs were eluted from the column with 2 ml methanol and 2 ml chloroform/methanol (2:1, v/v). The samples containing the eluate were evaporated under nitrogen for 1 h and dried under vacuum in an Eppendorf Concentrator at 30°C.

### GSL-Glycan Release by EGCase I, Reduction, and Purification

To release the glycans from the GSLs, a mixture of EGCase I (12 mU, 2 μl), EGCase I buffer (4 μl) and water (34 μl) (pH 5.2) was added to each sample and incubated for 36 h at 37°C. The released glycans were collected and loaded on TC18-RP-cartridges which had been preconditioned with 2 ml of methanol and 2 ml of water. The samples were washed with 200 ul of water and residual glycans were loaded to the cartridge. Then, 500 μl of water was added to the cartridge to wash the glycans from the column. The flow-through and wash fractions were pooled and dried in an Eppendorf Concentrator at 30°C.

The reduction was carried out with slight modifications following the same procedure as described in previous work (82). In brief, GSL-glycans were reduced to alditols in 20 μl of sodium borohydride (500 mM) in potassium hydroxide (50 mM) for 2 h at 50°C. Subsequently, 2 μl of 100% glacial acetic acid was added to neutralize the solution and quench the reaction. The desalting of GSL-glycans was performed as previously described Jensen et al. (82). Glycan alditols were eluted with 50 μl of water twice. The combined flow-through and eluate were pooled and dried under vacuum in an Eppendorf Concentrator at 30°C. The carbon SPE clean-up was performed and the purified glycan alditols were re-suspended in 20 μl of water prior to PGC nano-LC-ESI-MS/MS analysis.

### Exoglycosidase α2-3 Neuraminidase Treatment

Briefly, *O*-glycans and GSL-glycans alditols were resuspended in 18 μL of Glycobuffer 1 (New England Biolabs, Ipswich, MA, USA), and split into two aliquots, one for exoglycosidase α2–3 neuraminidase digestion and the other one was used as a control. Two μL of α2-3 neuraminidase was added to the samples for digestion, and 2 μL of Glycobuffer 1 to the controls. The samples were incubated overnight at 37°C and cleaned by PGC SPE, as described earlier Jensen et al. (82).

### PGC Nano-LC-ESI-MS/MS Analysis of *O*-Glycans and GSL-Glycans Alditols

The LC-MS/MS analysis of *O*-glycan and GSL-glycan alditols was performed on a Dionex Ultimate 3000 nano-LC system equipped with a Hypercarb PGC trap column (5 μm Hypercarb Kappa, 32 μm × 30 mm, Thermo Fisher Scientific, Waltham, MA) and a Hypercarb PGC nano-column (3 μm Hypercarb Kappa, 75 μm × 100 mm, Thermo Fisher Scientific, Waltham, MA) coupled to an amaZon speed ion trap mass spectrometer (Bruker Daltonics, Bremen, Germany). Mobile phase A consisted of 10 mM ammonium bicarbonate. Mobile phase B was 60% (v/v) acetonitrile/10 mM ammonium bicarbonate. To analyze glycans, 2 μl injections were performed and separation was achieved with a gradient of B (1–51% at 0.5%/min for *O*-glycan and 1–71% at 0.7%/min for GSL-glycans) followed by a 10-min wash step using 95% of B at a flow of rate of 0.6 μl/min. MS scans from m/z 340 to 1800 were recorded in enhanced mode using negative ion mode. MS/MS spectra were recorded using the top 3 highest intensity peaks.

Powerful isomer separation of glycans on PGC was performed. Structures of detected glycans were studied by fragmentation analysis using MS/MS in negative mode as well as additional exoglycosidase α2-3 neuraminidase treatment (data not shown). Glycan structures were assigned on the basis of the known MS/MS fragmentation patterns in negative-ion mode (28–30), signal patterns evidence from the profiling spectra, and general glycobiological knowledge, with help of Glycoworkbench (83) and Glycomod (84) software. The total quantity of glycans released from the same number of PaTu-S and PaTu-T cells was calculated by summing peak area of all detected glycans. The relative abundances of total *O*-glycans and GSL-glycans were determined by normalizing the total peak area of detected glycans in PaTu-S to 100%. Relative quantification of individual glycans was performed by normalizing the total peak area of all glycans within one sample normalizing it to 100%. Annotated glycan structures were used for calculations of structural glycan features. Relative abundances of specific glycan structures were grouped by summing relative abundances of each glycan multiplied by the number of epitopes per glycan. The glycans representing 95% of the relative abundance were identified and quantified. The bioinformatics analysis of *O*-glycans was performed following the standardized *e*-infrastructure, which has been submitted into the public repository UniCarb-DR (85).

### Flow Cytometry

PaTu-S and PaTu-T cells were incubated with anti-glycan antibodies for 1 h at 37°C in TSM (20 mM Tris-HCl, pH 7.4, 150 mM NaCl, 1 mM CaCl_2_ and 2 mM MgCl_2_), followed by a 30 min incubation with the fluorescent secondary antibody. Biotinylated lectins and human Gal-3 (5 μg/ml) were detected using Streptadivin AF 488 (Molecular Probes, Invitrogen). Recombinant human Gal-1 and human Gal-4 proteins (5 μg/ml) were incubated for 1 h at 4°C prior to antibody staining with anti-Gal-1 and anti-Gal-4 Abs, respectively. The lectins MGL-Fc (10 μg/ml), DC-SIGN-Fc (20 μg/ml), DCIR-Fc (100 ug/ml) and Dectin-1-Fc (20 μg/ml) were used in the presence or absence of 10 mM EDTA and detected using FITC-labeled anti-human-IgGFc. Fluorescence was measured using a FACScan flow cytometer (Becton Dickinson, Oxnard, CA), or a FACSCalibur flow cytometer. CellQuest (BD Biosciences, San Jose, CA) or Summit software was used to determine the mean fluorescence intensity (MFI) of cell populations, after which the background fluorescence negative control (cells stained only with the secondary antibody) was subtracted from the absolute MFI values.

### Cell Adhesion Assay

PaTu-S and PaTu-T were seeded onto a 96 well-plate at confluency. Cells were allowed to adhere overnight at 37°C + 5% CO_2_ in DMEM complete medium. Binding of Calceine AM-labeled iDCs to PaTu-S and PaTu-T cells was measured in TSM with 1% BSA. Briefly, 40.000 iDCs in the presence or absence of EGTA (10 mM), were added to each well-containing either PaTu-T or PaTu-S cells and were allowed to bind for 90 min at 37°C. Subsequently, non-adherent cells were removed by gentle washing. Cells were first visualized using a Leica DMILM (Leica Microsystems Wetzlar GmbH, Rijswijk, The Netherlands) and subsequently lysed, whereafter fluorescence was measured using a FLUOstar Spectrofluorimeter (BGM Labtech, Offenburg, Germany).

### Data and Statistical Analysis

Data are presented as mean ± SEM. Statistical analysis applied to the cell adhesion assay data was one-way ANOVA using Dunnett *t*-tests and for enzyme activity studies the bivariate student *t*-tests. Statistics on flow cytometry studies were performed using paired *t*-tests. Data were considered significant if *p* ≤ 0.05.

## Data Availability Statement

The microarray datasets for this study can be found in the Functional Glycomics Gateway (http://www.functionalglycomics.org/glycomics/publicdata/microarray.jsp), Accession 2087.

## Author Contributions

TZ, AB, SV, LL, JZ, and PD performed the experiments. TZ, BT, MW, AB, and ID conceptually designed the work. TZ, MW, ID, and AB wrote the manuscript. All authors read and commented on the manuscript.

## Conflict of Interest

The authors declare that the research was conducted in the absence of any commercial or financial relationships that could be construed as a potential conflict of interest.

## References

[1] Siegel RL Miller KD Jemal A Cancer statistics, 2018. CA Cancer J Clin. (2018) 68:7–30. 10.3322/caac.21442 29313949

[2] Pinho SS Reis CA. Glycosylation in cancer: mechanisms and clinical implications. Nat Rev Cancer. (2015) 15:540–55. 10.1038/nrc3982 26289314

[3] Engle DD Tiriac H Rivera KD Pommier A Whalen S Oni TE. The glycan CA19–9 promotes pancreatitis and pancreatic cancer in mice. Science. (2019) 364:1156–62. 10.1158/1538-7445.PANCA19-PR12 31221853 PMC6705393

[4] Munkley J. The glycosylation landscape of pancreatic cancer. Oncol Lett. (2019) 17:2569–75. 10.3892/ol.2019.9885 30854032 PMC6388511

[5] Wang M Zhu J Lubman DM Gao C. Aberrant glycosylation and cancer biomarker discovery: a promising and thorny journey. Clin Chem Lab Med. (2019) 57:407–16. 10.1515/cclm-2018-0379 30138110 PMC6785348

[6] Fuster MM Esko JD. The sweet and sour of cancer: glycans as novel therapeutic targets. Nat Rev Cancer. (2005) 5:526–42. 10.1038/nrc1649 16069816

[7] Reis CA Osorio H Silva L Gomes C David L. Alterations in glycosylation as biomarkers for cancer detection. J Clin Pathol. (2010) 63:322–9. 10.1136/jcp.2009.071035 20354203

[8] Tang H Partyka K Hsueh P Sinha JY Kletter D Zeh H. Glycans related to the CA19–9 antigen are elevated in distinct subsets of pancreatic cancers and improve diagnostic accuracy over CA19–9. Cell Mol Gastroenterol Hepatol. (2016) 2:201–21.e215. 10.1016/j.jcmgh.2015.12.003 26998508 PMC4792034

[9] Melo SA Luecke LB Kahlert C Fernandez AF Gammon ST Kaye J. Glypican-1 identifies cancer exosomes and detects early pancreatic cancer. Nature. (2015) 523:177–82. 10.1038/nature14581 26106858 PMC4825698

[10] Gill DJ Chia J Senewiratne J Bard F. Regulation of O-glycosylation through Golgi-to-ER relocation of initiation enzymes. J Cell Biol. (2010) 189:843–58. 10.1083/jcb.201003055 20498016 PMC2878949

[11] Zhang T de Waard AA Wuhrer M Spaapen RM The role of glycosphingolipids in immune cell functions. Front Immunol. (2019) 10:90 10.3389/fimmu.2019.00090 30761148 PMC6361815

[12] Park JH Nishidate T Kijima K Ohashi T Takegawa K Fujikane T. Critical roles of mucin 1 glycosylation by transactivated polypeptide N-acetylgalactosaminyltransferase 6 in mammary carcinogenesis. Cancer Res. (2010) 70:2759–69. 10.1158/0008-5472.CAN-09-3911 20215525

[13] Taniuchi K Cerny RL Tanouchi A Kohno K Kotani N Honke K. Overexpression of GalNAc-transferase GalNAc-T3 promotes pancreatic cancer cell growth. Oncogene. (2011) 30:4843–54. 10.1038/onc.2011.194 21625220 PMC3373266

[14] Miyoshi E Moriwaki K Nakagawa T. Biological function of fucosylation in cancer biology. J Biochem. (2008) 143:725–9. 10.1093/jb/mvn011 18218651

[15] Marcos NT Bennett EP Gomes J Magalhaes A Gomes C David L. ST6GalNAc-I controls expression of sialyl-Tn antigen in gastrointestinal tissues. Front Biosci. (2011) 3:1443–55. 10.2741/e345 21622148

[16] Harduin-Lepers A Krzewinski-Recchi MA Colomb F Foulquier F Groux-Degroote S Delannoy P. Sialyltransferases functions in cancers. Front Biosci. (2012) 4:499–515. 10.2741/e396 22201891

[17] Radhakrishnan P Dabelsteen S Madsen FB Francavilla C Kopp KL Steentoft C. Immature truncated O-glycophenotype of cancer directly induces oncogenic features. Proc Natl Acad Sci USA. (2014) 111:E4066–75. 10.1073/pnas.1406619111 25118277 PMC4191756

[18] Hofmann BT Schluter L Lange P Mercanoglu B Ewald F Folster A. COSMC knockdown mediated aberrant O-glycosylation promotes oncogenic properties in pancreatic cancer. Mol Cancer. (2015) 14:109. 10.1186/s12943-015-0386-1 26021314 PMC4447007

[19] Chugh S Meza J Sheinin YM Ponnusamy MP Batra SK. Loss of N-acetylgalactosaminyltransferase 3 in poorly differentiated pancreatic cancer: augmented aggressiveness and aberrant ErbB family glycosylation. Br J Cancer. (2016) 114:1376–86. 10.1038/bjc.2016.116 27187683 PMC4984453

[20] Elsasser HP Lehr U Agricola B Kern HF. Establishment and characterisation of two cell lines with different grade of differentiation derived from one primary human pancreatic adenocarcinoma. Virchows Arch B Cell Pathol Incl Mol Pathol. (1992) 61:295–306. 10.1007/BF02890431 1348891

[21] Marques IJ Weiss FU Vlecken DH Nitsche C Bakkers J Lagendijk AK. Metastatic behaviour of primary human tumours in a zebrafish xenotransplantation model. BMC Cancer. (2009) 9:128. 10.1186/1471-2407-9-128 19400945 PMC2697170

[22] Belo AI van der Sar AM Tefsen B van Die I. Galectin-4 reduces migration and metastasis formation of pancreatic cancer cells. PLoS ONE. (2013) 8:e65957. 10.1371/journal.pone.0065957 23824659 PMC3688853

[23] Maftouh M Belo AI Avan A Funel N Peters GJ Giovannetti E. Galectin-4 expression is associated with reduced lymph node metastasis and modulation of Wnt/beta-catenin signalling in pancreatic adenocarcinoma. Oncotarget. (2014) 5:5335–49. 10.18632/oncotarget.2104 24977327 PMC4170638

[24] Holst S Belo AI Giovannetti E van Die I Wuhrer M. Profiling of different pancreatic cancer cells used as models for metastatic behaviour shows large variation in their N-glycosylation. Sci Rep. (2017) 7:16623. 10.1038/s41598-017-16811-6 29192278 PMC5709460

[25] Belo AI van Vliet SJ Maus A Laan LC Nauta TD Koolwijk P. Hypoxia inducible factor 1alpha down regulates cell surface expression of alpha1,2-fucosylated glycans in human pancreatic adenocarcinoma cells. FEBS Lett. (2015) 589:2359–66. 10.1016/j.febslet.2015.07.035 26232512

[26] Ikehara Y Kojima N Kurosawa N Kudo T Kono M Nishihara S. Cloning and expression of a human gene encoding an N-acetylgalactosamine-alpha2,6-sialyltransferase. (ST6GalNAc I): a candidate for synthesis of cancer-associated sialyl-Tn antigens. Glycobiology. (1999) 9:1213–24. 10.1093/glycob/9.11.1213 10536037

[27] Ju T Lanneau GS Gautam T Wang Y Xia B Stowell SR. Human tumor antigens Tn and sialyl Tn arise from mutations in Cosmc. Cancer Res. (2008) 68:1636–46. 10.1158/0008-5472.CAN-07-2345 18339842

[28] Karlsson NG Schulz BL Packer NH. Structural determination of neutral O-linked oligosaccharide alditols by negative ion LC-electrospray-MSn. J Am Soc Mass Spectrom. (2004) 15:659–72. 10.1016/j.jasms.2004.01.002 15121195

[29] Karlsson NG Wilson NL Wirth HJ Dawes P Joshi H Packer NH. Negative ion graphitised carbon nano-liquid chromatography/mass spectrometry increases sensitivity for glycoprotein oligosaccharide analysis. Rapid Commun Mass Spectrom. (2004) 18:2282–92. 10.1002/rcm.1626 15384149

[30] Anugraham M Everest-Dass AV Jacob F Packer NH. A platform for the structural characterization of glycans enzymatically released from glycosphingolipids extracted from tissue and cells. Rapid Commun Mass Spectrom. (2015) 29:545–61. 10.1002/rcm.7130 26212272

[31] Rabinovich GA van Kooyk Y Cobb BA. Glycobiology of immune responses. Ann N Y Acad Sci. (2014) 1253:1–15. 10.1111/j.1749-6632.2012.06492.x 22524422 PMC3884643

[32] Demirkan B. The roles of epithelial-to-mesenchymal transition (EMT) and mesenchymal-to-epithelial transition. (MET) in breast cancer bone metastasis: potential targets for prevention and treatment. J Clin Med. (2013) 2:264–82. 10.3390/jcm2040264 26237148 PMC4470149

[33] Tsai JH Yang J. Epithelial-mesenchymal plasticity in carcinoma metastasis. Genes Dev. (2013) 27:2192–206. 10.1101/gad.225334.113 24142872 PMC3814640

[34] Mittal V Epithelial mesenchymal transition in tumor metastasis. Annu Rev Pathol. (2018) 13:395–412. 10.1146/annurev-pathol-020117-043854 29414248

[35] Drabsch Y Snaar-Jagalska BE Ten Dijke P. Fish tales: the use of zebrafish xenograft human cancer cell models. Histol Histopathol. (2017) 32:673–86. 10.14670/HH-11-853 27933602

[36] Moniaux N Andrianifahanana M Brand RE Batra SK. Multiple roles of mucins in pancreatic cancer, a lethal and challenging malignancy. Br J Cancer. (2004) 91:1633–8. 10.1038/sj.bjc.6602163 15494719 PMC2409950

[37] Kaur S Kumar S Momi N Sasson AR Batra SK. Mucins in pancreatic cancer and its microenvironment. Nat Rev Gastroenterol Hepatol. (2013) 10:607–20. 10.1038/nrgastro.2013.120 23856888 PMC3934431

[38] Ju T Cummings RD. A unique molecular chaperone Cosmc required for activity of the mammalian core 1 beta 3-galactosyltransferase. Proc Natl Acad Sci USA. (2002) 99:16613–8. 10.1073/pnas.262438199 12464682 PMC139192

[39] Mi R Song L Wang Y Ding X Zeng J Lehoux S. Epigenetic silencing of the chaperone cosmc in human leukocytes expressing Tn antigen. J BiolChem. (2012) 287:41523–33. 10.1074/jbc.M112.371989 23035125 PMC3510848

[40] Chugh S Barkeer S Rachagani S Nimmakayala RK Perumal N Pothuraju R. Disruption of C1galt1 gene promotes development and metastasis of pancreatic adenocarcinomas in mice. Gastroenterology. (2018) 155:1608–24. 10.1053/j.gastro.2018.08.007 30086262 PMC6219903

[41] Gu C Oyama T Osaki T Li J Takenoyama M Izumi H. Low expression of polypeptide GalNAc N-acetylgalactosaminyl transferase-3 in lung adenocarcinoma: impact on poor prognosis and early recurrence. Br J Cancer. (2004) 90:436–42. 10.1038/sj.bjc.6601531 14735190 PMC2409559

[42] Maupin KA Sinha A Eugster E Miller J Ross J Paulino V. Glycogene expression alterations associated with pancreatic cancer epithelial-mesenchymal transition in complementary model systems. PLoS ONE. (2010) 5:e13002. 10.1371/journal.pone.0013002 20885998 PMC2946336

[43] Sahasrabudhe NM Lenos K van der Horst JC Rodriguez E van Vliet SJ. Oncogenic BRAFV600E drives expression of MGL ligands in the colorectal cancer cell line HT29 through N-acetylgalactosamine-transferase 3. Biol Chem. (2018) 399:649–59. 10.1515/hsz-2018-0120 29894293

[44] Peng R-Q Wan H-Y Li H-F Liu M Li X Tang H. MicroRNA-214 suppresses growth and invasiveness of cervical cancer cells by targeting UDP-N-acetyl-α-d-galactosamine: polypeptide N-acetylgalactosaminyltransferase 7. J Biol Chem. (2012) 287:14301–9. 10.1074/jbc.M111.337642 22399294 PMC3340176

[45] Gaziel-Sovran A Segura MF Di Micco R Collins MK Hanniford D Vega-Saenz de Miera E. miR-30b/30d regulation of GalNAc transferases enhances invasion and immunosuppression during metastasis. Cancer Cell. (2011) 20:104–18. 10.1016/j.ccr.2011.05.027 21741600 PMC3681522

[46] Hattori H Uemura K Ishihara H Ogata H. Glycolipid of human pancreatic cancer; the appearance of neolacto-series. (type 2 chain) glycolipid and the presence of incompatible blood group antigen in tumor tissues. Biochim Biophys Acta. (1992) 1125:21–7. 10.1016/0005-2760(92)90150-T 1567904

[47] Satoh M Handa K Saito S Tokuyama S Ito A Miyao N. Disialosyl galactosylgloboside as an adhesion molecule expressed on renal cell carcinoma and its relationship to metastatic potential. Cancer Res. (1996) 56:1932–8. 8620516

[48] Mandal C Sarkar S Chatterjee U Schwartz-Albiez R Mandal C. Disialoganglioside GD3-synthase over expression inhibits survival and angiogenesis of pancreatic cancer cells through cell cycle arrest at S-phase and disruption of integrin-beta1-mediated anchorage. Int J Biochem Cell Biol. (2014) 53:162–73. 10.1016/j.biocel.2014.05.015 24842107

[49] Inokuchi J Jimbo M Momosaki K Shimeno H Nagamatsu A Radin NS. Inhibition of experimental metastasis of murine lewis lung carcinoma by an inhibitor of glucosylceramide synthase and its possible mechanism of action. Cancer Res. (1990) 50:6731–7. 2145065

[50] Liu Y-Y Han T-Y Giuliano AE Cabot MC. Ceramide glycosylation potentiates cellular multidrug resistance. Faseb J. (2001) 15:719–30. 10.1096/fj.00-0223com 11259390

[51] Gupta V Bhinge KN Hosain SB Xiong K Gu X Shi R. Ceramide glycosylation by glucosylceramide synthase selectively maintains the properties of breast cancer stem cells. J Biol Chem. (2012) 287:37195–205. 10.1074/jbc.M112.396390 22936806 PMC3481319

[52] Kovbasnjuk O Mourtazina R Baibakov B Wang T Elowsky C Choti MA. The glycosphingolipid globotriaosylceramide in the metastatic transformation of colon cancer. Proc Natl Acad Sci USA. (2005) 102:19087–92. 10.1073/pnas.0506474102 16365318 PMC1323164

[53] Falguieres T Maak M von Weyhern C Sarr M Sastre X Poupon MF. Human colorectal tumors and metastases express Gb3 and can be targeted by an intestinal pathogen-based delivery tool. Mol Cancer Ther. (2008) 7:2498–508. 10.1158/1535-7163.MCT-08-0430 18687997

[54] Stimmer L Dehay S Nemati F Massonnet G Richon S Decaudin D. Human breast cancer and lymph node metastases express Gb3 and can be targeted by STxB-vectorized chemotherapeutic compounds. BMC Cancer. (2014) 14:916. 10.1186/1471-2407-14-916 25476116 PMC4289340

[55] Geyer PE Maak M Nitsche U Perl M Novotny A Slotta-Huspenina J. Gastric adenocarcinomas express the glycosphingolipid Gb3/CD77: targeting of gastric cancer cells with shiga toxin B-subunit. Mol Cancer Ther. (2016) 15:1008–17. 10.1158/1535-7163.MCT-15-0633 26826119

[56] Storck W Meisen I Gianmoena K Plager I Kouzel IU Bielaszewska M. Shiga toxin glycosphingolipid receptor expression and toxin susceptibility of human pancreatic ductal adenocarcinomas of differing origin and differentiation. Biol Chem. (2012) 393:785–99. 10.1515/hsz-2012-0165 22944681

[57] Balmana M Gimenez E Puerta A Llop E Figueras J Fort E. Increased alpha1–3 fucosylation of alpha-1-acid glycoprotein. (AGP) in pancreatic cancer. J Proteomics. (2016) 132:144–54. 10.1016/j.jprot.2015.11.006 26563517

[58] Morishita K Ito N Koda S Maeda M Nakayama K Yoshida K. Haptoglobin phenotype is a critical factor in the use of fucosylated haptoglobin for pancreatic cancer diagnosis. Clin Chim Acta. (2018) 487:84–9. 10.1016/j.cca.2018.09.001 30189188

[59] Miyoshi E Moriwaki K Terao N Tan CC Terao M Nakagawa T. Fucosylation is a promising target for cancer diagnosis and therapy. Biomolecules. (2012) 2:34–45. 10.3390/biom2010034 24970126 PMC4030867

[60] Schultz M Swindall A Bellis S. Regulation of the metastatic cell phenotype by sialylated glycans. Cancer Metastasis Rev. (2012) 31:501–18. 10.1007/s10555-012-9359-7 22699311 PMC4079276

[61] Perez-Garay M Arteta B Pages L de Llorens R de Bolos C Vidal-Vanaclocha F. alpha2,3-sialyltransferase ST3Gal III modulates pancreatic cancer cell motility and adhesion in vitro and enhances its metastatic potential *in vivo*. PLoS ONE. (2010) 5:12524. 10.1371/journal.pone.0012524 20824144 PMC2931708

[62] Bassaganas S Carvalho S Dias AM Perez-Garay M Ortiz MR Figueras J. Pancreatic cancer cell glycosylation regulates cell adhesion and invasion through the modulation of alpha2beta1 integrin and E-cadherin function. PLoS ONE. (2014) 9:e98595. 10.1371/journal.pone.0098595 24878505 PMC4039506

[63] Bassaganas S Perez-Garay M Peracaula R. Cell surface sialic acid modulates extracellular matrix adhesion and migration in pancreatic adenocarcinoma cells. Pancreas. (2014) 43:109–17. 10.1097/MPA.0b013e31829d9090 23921962

[64] Appelmelk BJ van Die I van Vliet SJ Vandenbroucke-Grauls CM Geijtenbeek TB van Kooyk Y. Cutting edge: carbohydrate profiling identifies new pathogens that interact with dendritic cell-specific ICAM-3-grabbing nonintegrin on dendritic cells. J Immunol. (2003) 170:1635–9. 10.4049/jimmunol.170.4.1635 12574325

[65] van Vliet SJ van Liempt E Saeland E Aarnoudse CA Appelmelk B Irimura T. Carbohydrate profiling reveals a distinctive role for the C-type lectin MGL in the recognition of helminth parasites and tumor antigens by dendritic cells. Int Immunol. (2005) 17:661–9. 10.1093/intimm/dxh246 15802303

[66] van Vliet SJ Bay S Vuist IM Kalay H Garcia-Vallejo JJ Leclerc C. MGL signaling augments TLR2-mediated responses for enhanced IL-10 and TNF-alpha secretion. J Leukoc Biol. (2013) 94:315–23. 10.1189/jlb.1012520 23744646

[67] Yuan M Zhang X Zhang J Wang K Zhang Y Shang W. DC-SIGN-LEF1/TCF1-miR-185 feedback loop promotes colorectal cancer invasion and metastasis. Cell Death Differ. (2019) 27:379–95. 10.1038/s41418-019-0361-2 31217502 PMC7205996

[68] Marcelo F Supekar N Corzana F van der Horst JC Vuist IM Live D. Identification of a secondary binding site in human macrophage galactose-type lectin by microarray studies: implications for the molecular recognition of its ligands. J Biol Chem. (2019) 294:1300–11. 10.1074/jbc.RA118.004957 30504228 PMC6349122

[69] Ideo H Seko A Ohkura T Matta KL Yamashita K. High-affinity binding of recombinant human galectin-4 to SO(3)(-)–>3Galbeta1–>3GalNAc pyranoside. Glycobiology. (2002) 12:199–208. 10.1093/glycob/12.3.199 11971864

[70] Mereiter S Balmana M Campos D Gomes J Reis CA. Glycosylation in the Era of cancer-targeted therapy: where are we heading? Cancer Cell. (2019) 36:6–16. 10.1016/j.ccell.2019.06.006 31287993

[71] Dias-Baruffi M Stowell SR Song SC Arthur CM Cho M Rodrigues LC. Differential expression of immunomodulatory galectin-1 in peripheral leukocytes and adult tissues and its cytosolic organization in striated muscle. Glycobiology. (2010) 20:507–20. 10.1093/glycob/cwp203 20053628 PMC2900886

[72] Toscano MA Commodaro AG Ilarregui JM Bianco GA Liberman A Serra HM. Galectin-1 suppresses autoimmune retinal disease by promoting concomitant Th2- and T regulatory-mediated anti-inflammatory responses. J Immunol. (2006) 176:6323–32. 10.4049/jimmunol.176.10.6323 16670344

[73] van Stijn CM van den Broek M van de Weerd R Visser M Tasdelen I Tefsen B. Regulation of expression and secretion of galectin-3 in human monocyte-derived dendritic cells. Mol Immunol. (2009) 46:3292–9. 10.1016/j.molimm.2009.07.026 19699526

[74] van Vliet SJ Gringhuis SI Geijtenbeek TB van Kooyk Y. Regulation of effector T cells by antigen-presenting cells via interaction of the C-type lectin MGL with CD45. Nat Immunol. (2006) 7:1200–8. 10.1038/ni1390 16998493

[75] Geijtenbeek TB van Duijnhoven GC van Vliet SJ Krieger E Vriend G Figdor CG. Identification of different binding sites in the dendritic cell-specific receptor DC-SIGN for intercellular adhesion molecule 3 and HIV-1. J Biol Chem. (2002) 277:11314–20 10.1074/jbc.M111532200 11799126

[76] Bloem K Vuist IM van den Berk M Klaver EJ van Die I Knippels LM. DCIR interacts with ligands from both endogenous and pathogenic origin. Immunol Lett. (2014) 158:33–41. 10.1016/j.imlet.2013.11.007 24239607

[77] Benjamini Y Hochberg Y Controlling the false discovery rate: a practical and powerful approach to multipletesting. J R Statist Soc Seri. (1995) 57:289–300. 10.1111/j.2517-6161.1995.tb02031.x

[78] Garcia-Vallejo JJ Gringhuis SI van Dijk W van Die I. Gene expression analysis of glycosylation-related genes by real-time polymerase chain reaction. Methods Mol Biol. (2006) 347:187–209. 10.1385/1-59745-167-3:187 17072012

[79] Ju T Xia B Aryal RP Wang W Wang Y Ding X. A novel fluorescent assay for T-synthase activity. Glycobiology. (2011) 21:352–62. 10.1093/glycob/cwq168 20959392 PMC3033746

[80] Bolscher JG Brevoord J Nazmi K Ju T Veerman EC van Wijk JA. Solid-phase synthesis of a pentavalent GalNAc-containing glycopeptide. (Tn antigen) representing the nephropathy-associated IgA hinge region. Carbohydr Res. (2010) 345:1998–2003. 10.1016/j.carres.2010.07.022 20719305 PMC2940223

[81] Palcic M Heerze L Pierce M Hindsgaul O The use of hydrophobic synthetic glycosides as acceptors in glycosyltransferase assays. Glycoconjugate J. (1988) 5:49–63. 10.1007/BF01048331

[82] Jensen PH Karlsson NG Kolarich D Packer NH. Structural analysis of N- and O-glycans released from glycoproteins. Nat Protoc. (2012) 7:1299–310. 10.1038/nprot.2012.063 22678433

[83] Ceroni A Maass K Geyer H Geyer R Dell A Haslam SM. GlycoWorkbench: a tool for the computer-assisted annotation of mass spectra of glycans. J Proteome Res. (2008) 7:1650–9. 10.1021/pr7008252 18311910

[84] Cooper CA Gasteiger E Packer NH. GlycoMod–a software tool for determining glycosylation compositions from mass spectrometric data. Proteomics. (2001) 1:340–9. 10.1002/1615-9861(200102)1:2<340::AID-PROT340>3.0.CO;2-B 11680880

[85] Rojas-Macias MA Mariethoz J Andersson P Jin C Venkatakrishnan V Aoki NP. Towards a standardized bioinformatics infrastructure for N- and O-glycomics. Nat Commun. (2019) 10:3275. 10.1038/s41467-019-11131-x 31332201 PMC6796180

