# Supplementary material for: Differential O- and Glycosphingolipid Glycosylation in Human Pancreatic Adenocarcinoma Cells With Opposite Morphology and Metastatic Behavior
Source: Front Oncol. 2020 Jun 2;10:732. doi: 10.3389/fonc.2020.00732 (PMC7280451; doi:10.3389/fonc.2020.00732)
Supplement: Supplementary file 1 [file Data_Sheet_1.PDF]

Supplementary Material

1 Supplementary Figures and Tables

The supplementary Material for this article can be found online.

Supplementary Figure S1: Flow cytometry binding assay of anti-Tn mAb and PNA to PaTu-S and PaTu-T cells (Relates to **Figure 3C** and **3E**).

Supplementary Figure S2: Structural analysis of monosialylated *O*-glycan isomers of *m/z* 675.30 $[M-H]^-$ derived from PaTu-S and PaTu-T cells on PGC nano-LC-ESI-MS/MS in negative ion mode (Relates to **Figure 4**).

Supplementary Figure S3: Structural analysis of monosialylated GSL-glycan isomers of *m/z* 999.30 $[M-H]^-$ derived from PaTu-S and PaTu-T cells on PGC nano-LC-ESI-MS/MS in negative ion mode (Relates to **Figure 5**).

Supplementary Figure S4: Relative quantification of individual *O*-glycans derived from PaTu-S and PaTu-T cell lines on PGC nano-LC-ESI-MS/MS (Relates to **Figure 4**).

Supplementary Figure S5: Relative quantification of individual GSL-glycans derived from PaTu-S and PaTu-T cell lines on PGC nano-LC-ESI-MS/MS (Relates to **Figure 5**).

Supplementary Figure S6: Representative overlay histograms of plant lectin binding to PaTu-S and PaTu-T cells from at least three independent experiments (Relates to **Figure 6A**).

Supplementary Figure S7: Relative abundance of glycan structural classes in *O*-glycans and GSL-glycans derived from PaTu-S and PaTu-T cells on PGC nano-LC-ESI-MS/MS in negative ion mode.

Supplementary Table 1: Summary of glycosylation changes observed in pancreatic cancer epithelial-like PaTu-S and mesenchymal-like PaTu-T cell lines.

1.1 Supplementary Figures

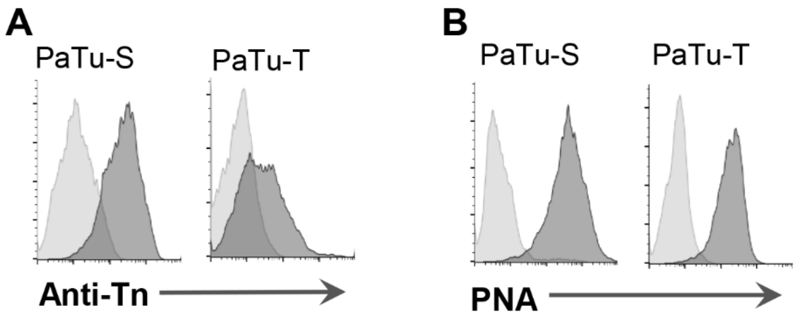

Supplementary Figure S1. Flow cytometry binding assay of anti-Tn mAb and PNA to PaTu-S and PaTu-T cells. Representative overlay histograms of the binding of (A) anti-Tn mAb and (B) PNA to PaTu-S and PaTu-T cells from at least three independent experiments are depicted. Dark grey field: staining with the antibody or lectin against the respective structure by means of fluorescent intensity; light grey field: background staining with secondary antibodies.

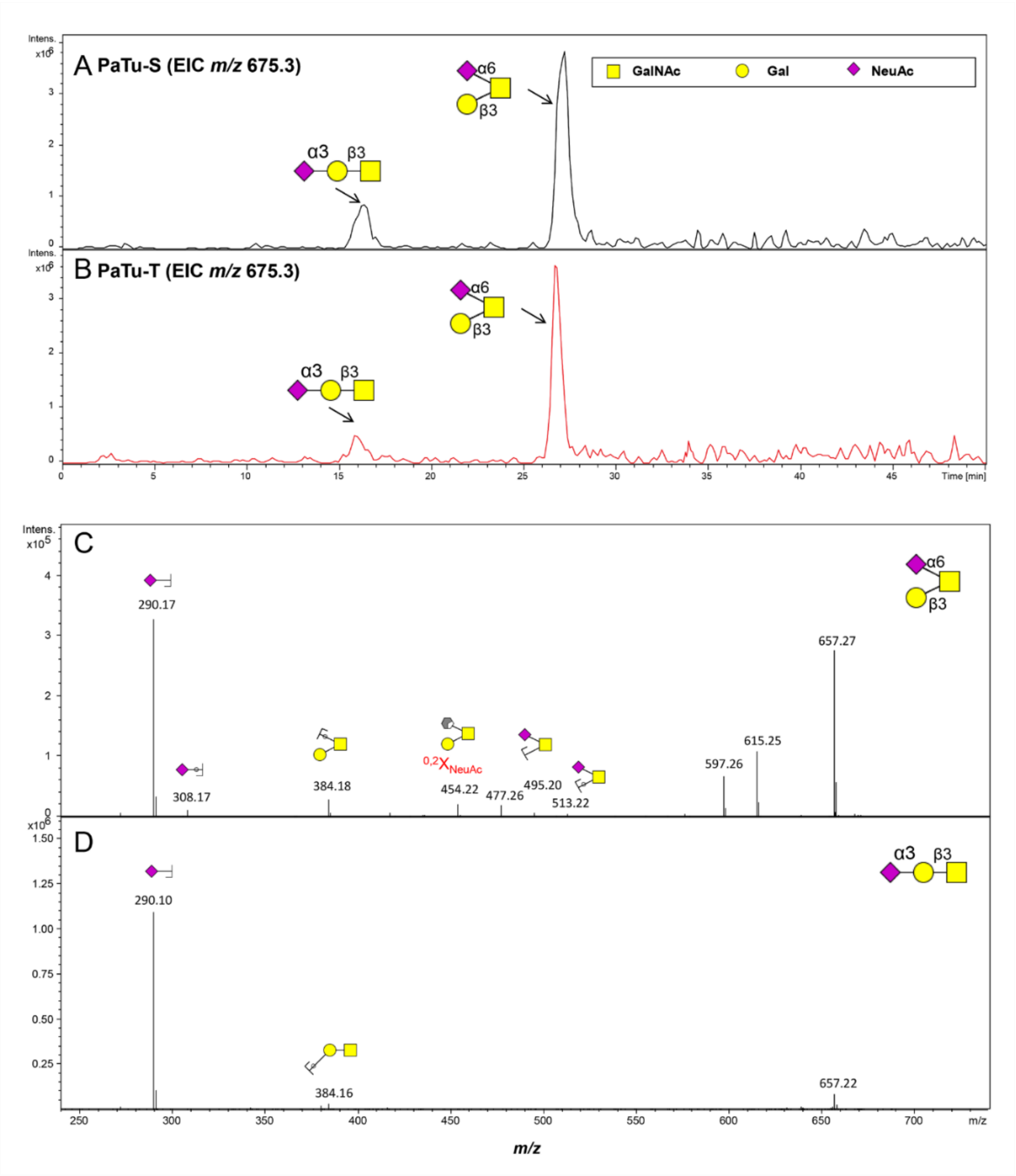

Supplementary Figure S2. Structural analysis of monosialylated *O*-glycan isomers of *m/z* 675.30 $[M\text{-}H]^-$. Chromatographic separation of monosialylated *O*-glycan isomers of *m/z* 675.30 $[M\text{-}H]^-$ in PaTu-S **(A)** and PaTu-T **(B)** cell lines. The extracted ion chromatograms show a different retention behaviour of monosialylated *O*-glycan isomers of *m/z* 675.30 $[M\text{-}H]^-$. MS/MS fragmentation spectrum of the two isomers from PaTu-S cell lines confirm the structures of α2-6 sialylated core GalNAc (**C,** RT= 16.1 min) and α2-3 sialylated T antigen (**D**, RT= 26.9 min). The specific fragment ion *m/z* 454.22 results from a $^{0,2}X_{NeuAc}$ cross-ring fragmentation of α2-6 sialic acid. The fragment Z ion *m/z* 495.20 and fragment Y ion *m/z* 513.22 in the α2-6 sialylated core GalNAc isomer indicate the sialic acid is linked on the core GalNAc, compared to the absence of these ions in the sialylated T antigen.

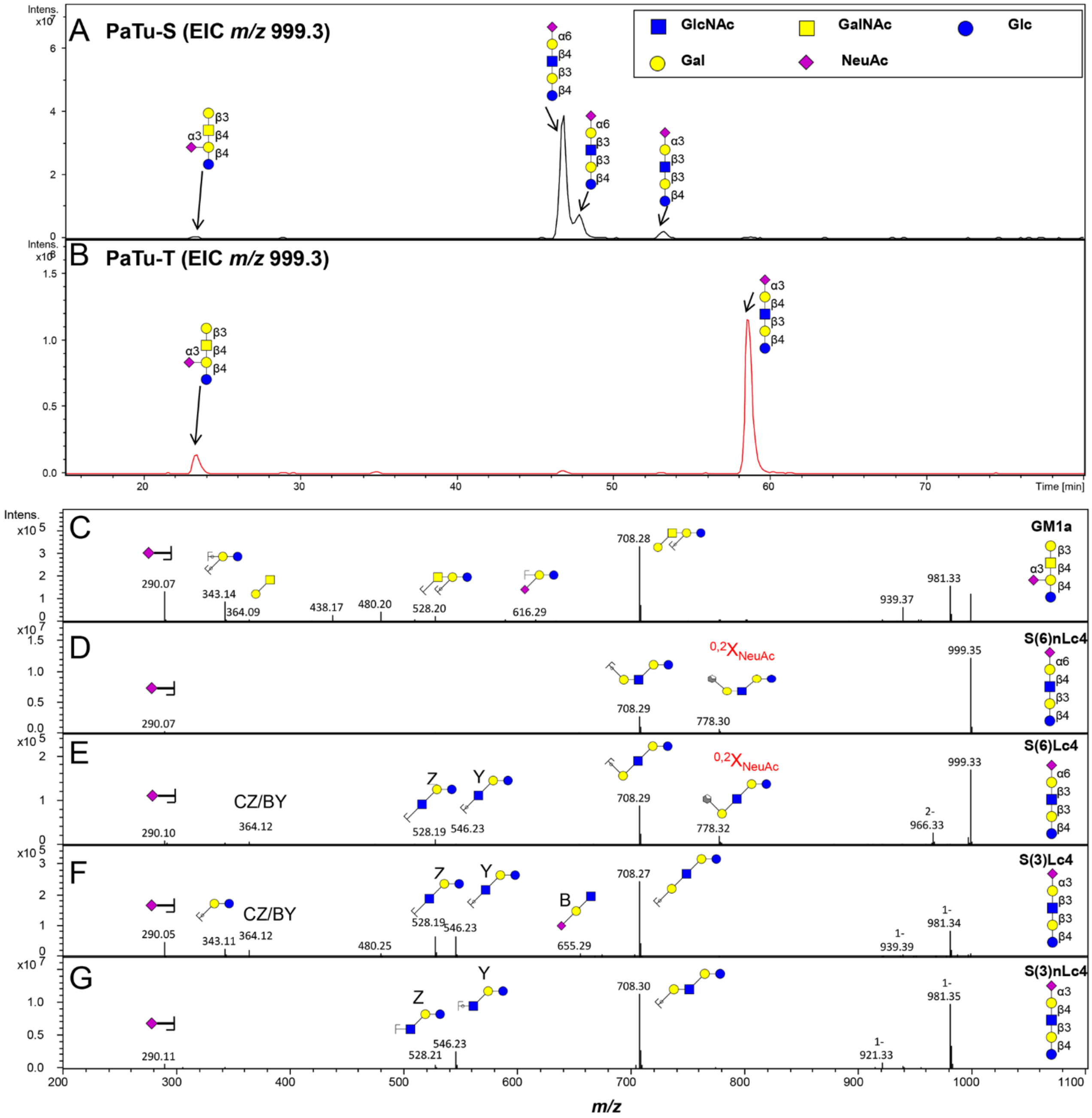

Supplementary Figure S3. Structural analysis of monosialylated GSL-glycan isomers of *m/z* 999.30 $[M-H]^-$ with the same composition. Chromatographic separation of monosialylated GSL-glycan isomers of *m/z* 999.30 $[M-H]^-$ in PaTu-S **(A)** and PaTu-T **(B)** cell lines. The extracted ion chromatograms show a different retention behaviour of five different isomers of *m/z* 999.30 $[M-H]^-$. MS/MS fragmentation spectrum of the four isomers from PaTu-S cell lines confirm the structures of GM1a (**C,** RT= 23.6min), S(6)nLc4 (**D,** RT= 46.4min), S(6)Lc4 (**E,** RT= 47.9min) and S(3)Lc4 (**F,** RT= 53.1 min), while the second isomer was assigned as S(3)nLc4 (**G,** RT= 58.9min) based on MS/MS fragmentation spectrum in PaTu-T cell line. The specific fragment ion *m/z* 616.30 in GM1a indicate a sialylated lactose, which is not present in the terminal sialylated neolacto-series GSL-glycans. The specific fragment ion *m/z* 778.30 result from a $^{0,2}X_{NeuAc}$ cross-ring fragmentation of α2-6 sialic acid, indicating the α2-6 sialic acid linked to terminal galactose.

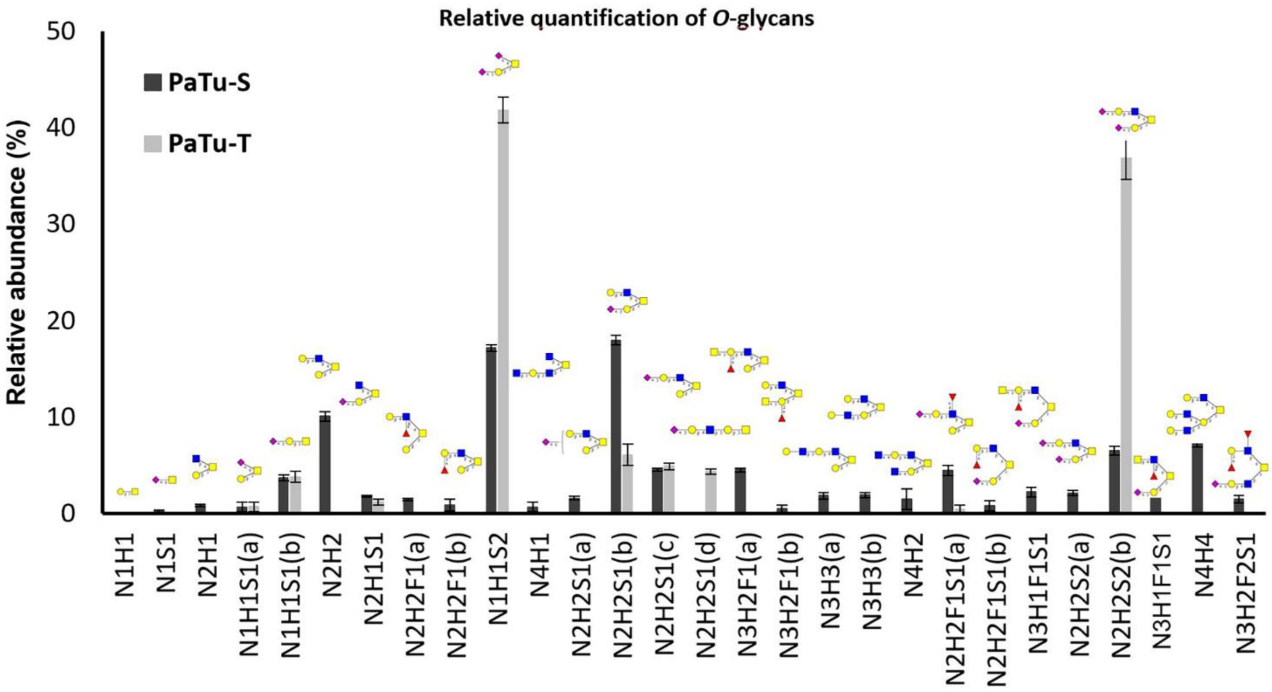

Supplementary Figure S4. Relative quantification of individual *O*-glycans derived from 0.5 million PaTu-S and PaTu-T cells on PGC nano-LC-ESI-MS/MS in negative ion mode (displayed as mean relative abundance plus standard deviation; N=3). Structures are depicted according to the CFG (Consortium of Functional Glycomics). Blue square: *N*-acetylglucosamine, yellow circle: galactose, red triangle: fucose, pink diamond: *N*-acetylneuraminic acid. Composition: H: hexose; N: *N*-acetylhexosamines; S: *N*-acetylneuraminic acid; a, b, c, d: isomer number.

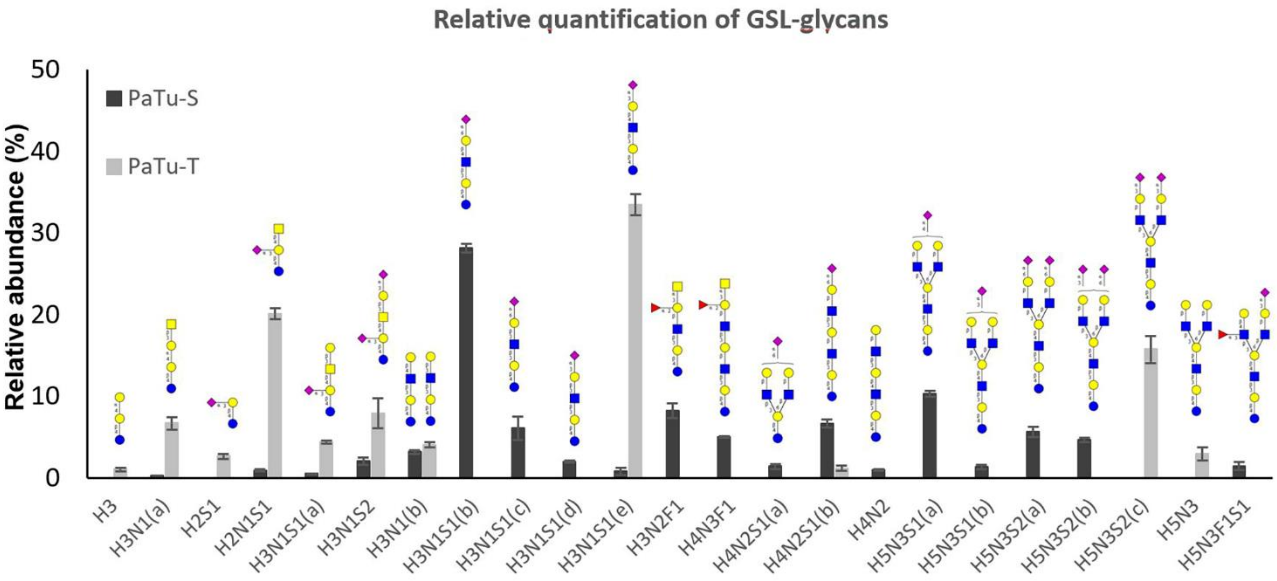

Supplementary Figure S5. Relative quantification of individual GSL-glycans derived from 2 million PaTu-S and PaTu-T cells on PGC nano-LC-ESI-MS/MS in negative ion mode (displayed as mean relative abundance plus standard deviation; N=3). Structures are depicted according to the CFG (Consortium of Functional Glycomics). Blue square: *N*-acetylglucosamine, yellow circle: galactose, red triangle: fucose, pink diamond: *N*-acetylneuraminic acid. Composition: H: hexose; N: *N*-acetylhexosamines; S: *N*-acetylneuraminic acid; a, b, c, d: isomer number.

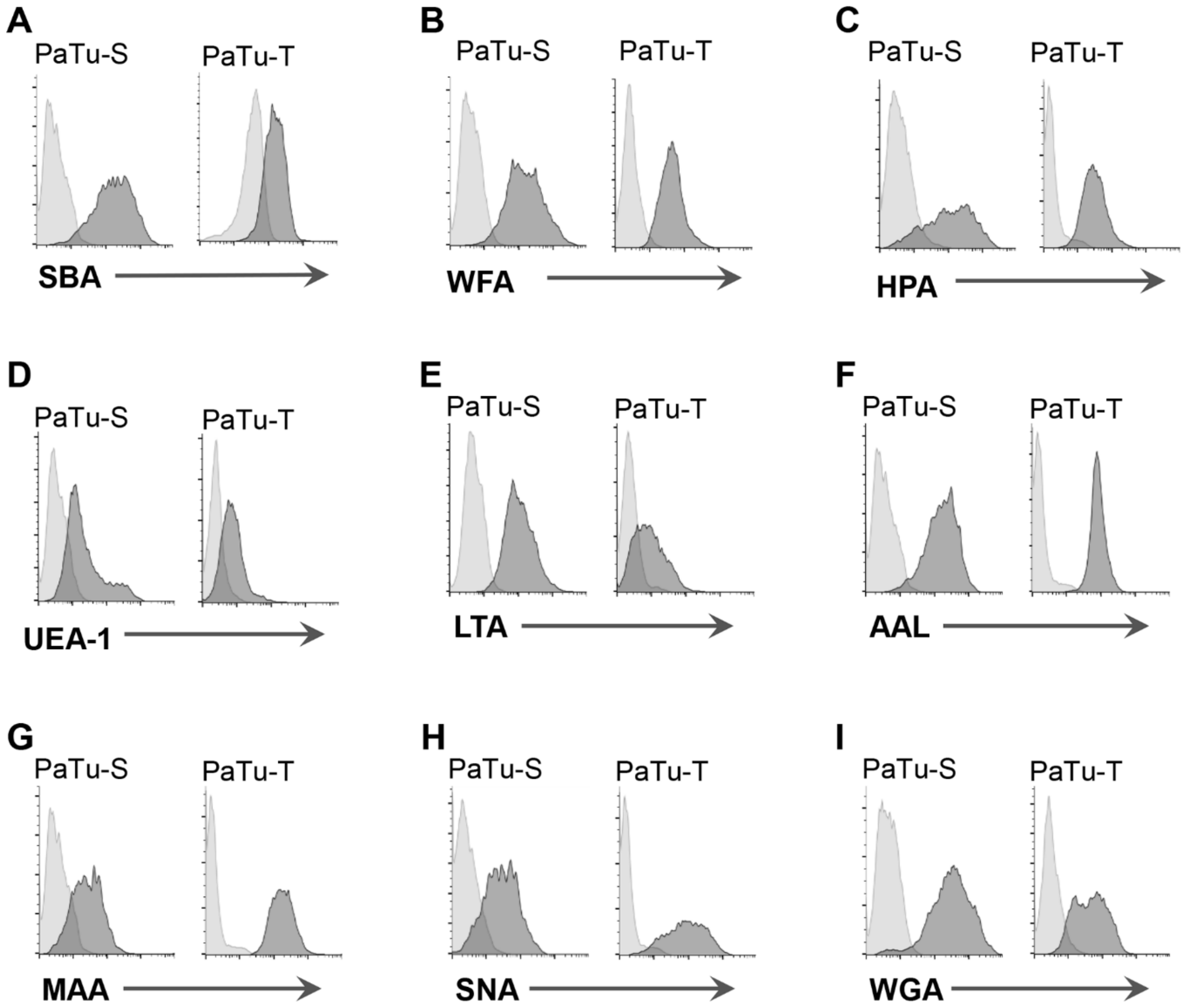

Supplementary Figure S6. Flow cytometry binding assay with plant lectins. Representative overlay histograms of the binding of lectins **(A)** SBA, **(B)** WFA, **(C)** HPA, **(D)** UEA-1, **(E)** LTA, **(F)** AAL, **(G)** MAA, **(H)** SNA and **(I)** WGA to PaTu-S and PaTu-T cells from at least three independent experiments are depicted. Dark grey field: staining with the lectin against the respective structure by means of fluorescent intensity; light grey field: background staining with secondary antibodies.

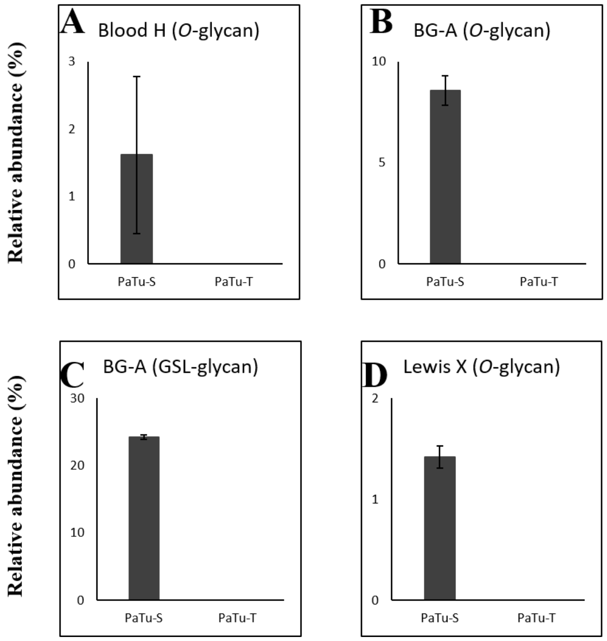

Supplementary Figure S7. Relative abundance of glycan structural classes in *O*-glycans and GSL-glycans derived from PaTu-S and PaTu-T cells on PGC nano-LC-ESI-MS/MS in negative ion mode (displayed as mean relative abundance plus standard deviation; N=3). **(A)** Blood group H antigen in *O*-glycans, **(B)** Blood group A (BG-A) in *O*-glycans, **(C)** BG-A in GSL-glycans, and **(D)** Lewis X in *O*-glycans.

Supplementary Table S1: Summary of glycosylation changes between pancreatic cancer epithelial-like PaTu-S and mesenchymal-like PaTu-T cell lines.

**Glycan types**	**Mesenchymal-like PaTu-T (T) compared to Epithelial-like PaTu-S (S)**		
		**Expression**	**Observation**
N-glycans	Oligomannose	T > S	MS: increased abundance of oligomannose<br>Binding assay: higher binding to GNA and ConA
	Complex	T < S	RNA array: increased *MAN2A1* and decreased *MAN2A2*<br>MS: decreased abundance of complex structure
	Hybrid	T < S	MS: decreased abundance of hybrid
	Branching	T < S	RNA array: decreased *MGAT4A*<br>MS: decreased abundance of tetra-antennary
O-glycans	Tn antigen	S	RNA array: lower expression of total *ppGANLTTs* (Decreased *GALNT3**, *4*, *5**, *6*, *7*, and *12**; increased *GALNT14* and *18*; minor change in *GALNT1* and *2*)<br>qPCR: Decreased *GALNT3**, *5**,and *12**; minor change in *GALNT2*, *7*, and *14*)<br>Binding assay: lower binding to MGL
	T antigen	T < S	RNA array: decreased *C1GALT1*<br>qPCR: decreased *C1GALT1*<br>Enzyme assay with increased T antigen<br>MS: absent of T antigen<br>Binding assay: lower binding to PNA
	Sialylated Tn	T < S	RNA array: decreased *ST6GANAC1*<br>MS: absent of Sialyl-T antigen
	Normalized total *O*-glycans	T < S	RNA array: lower expression of total *ppGANLTTs* (Decreased *GALNT3**, *4*, *5**, *6*, *7*, and *12**; increased *GALNT14* and *18*; minor change in *GALNT1* and *2*)<br>qPCR: Decreased *GALNT3**, *5**,and *12**; minor change in *GALNT2*, *7*, and *14*)<br>Enzyme assay: decreased Tn antigen;<br>MS: normalized total *O*-glycans
	Core 1	T < S	RNA array: lower expression of *C1GALT1*<br>qPCR: lower expression of *C1GALT1*<br>Enzyme assay with decreased T antigen<br>MS: increased abundance of total core 1 and depletion of T antigen
	Core 2/4	T < S	RNA array: decreased expression of *GCNT1* and *3*<br>Lower protein level of GCNT1 and GCNT3<br>MS: decreased abundance of core 2/4
	Core 3	T ≈ S	RNA array: no change in *B3GNT6*<br>MS: N.D.
	Core 1 α2-6 sialylation	T > S	RNA array: higher expression of *ST6GALNAC4* and *6*<br>qPCR: higher expression of *ST6GALNAC4* and *6*<br>MS: increased abundance of core GalNAc α2-6 sialylation<br>Binding assay: higher binding to SNA
GSL-glycans	Normalized total glucosylceramide	T < S	RNA array: higher expression of *UGCG*, *B4GALT5*, *6*, *ST3GAL5*,*A4GALT*, and *B4GALNT1*, lower expression of *UGCGL2* and *B3GNT5*<br>qPCR: higher expression of *UGCG*, *ST3GAL5*, and *A4GALT*, lower expression of *B3GNT5*

			MS: decreased normalized total abundance of GSL-glycans
	Ganglioside	T > S	RNA array: higher expression of *B4GALNT1* and *ST3GAL5*<br>qPCR: higher expression of *B4GALNT1* and specific expression of *ST3GAL5*<br>MS: increased abundance of ganglioside
	Globoside	T	RNA array: specific expression of *A4GALT*<br>qPCR: specific expression of *A4GALT*<br>MS: specific expression of globoside (Gb3 and Gb4)
	nsGSLs	T < S	RNA array: lower expression of *B3GNT5*<br>qPCR: lower expression of *B3GNT5*<br>MS: decreased abundance of nsGSLs
	Galactosylceramide	T < S	RNA array: decreased expression of *GAL3ST1*
Termination	α2-3 sialylation	T > S	RNA array: higher expression of *ST3GAL1*,*3*, *4*, and *6*<br>qPCR: higher expression of *ST3GAL3*, *4*, and *6*; minor change in *ST3GAL1*<br>MS: increased abundance in *N*-glycans, *O*-glycans and GSL-glycans<br>Binding assay: higher binding to MAA
	α2-6 sialylation	T > S	RNA array: higher expression of *ST6GALNAC4* and *6*; and decreased expression of *ST6GAL1*<br>qPCR: higher expression of *ST6GALNAC4* and *6*; and decreased expression of *ST6GAL1*<br>MS: increased abundance of core α2-6 sialylation on GalNAc in *O*-glycans but decreased α2-6 sialylation on Gal in *N*-, *O*-glycans and GSL-glycans<br>Binding assay: higher binding to SNA
	α1-2 fucosylation	T < S	RNA array: lower expression of *FUT1* and *2*<br>qPCR: lower expression of *FUT1* and *2*<br>MS: decreased total abundance of fucosylation in *N*-glycans, depletion of α1-2 fucosylation in *O*-glycans and GSL-glycans<br>Binding assay: lower binding to UEA-1 and DC-SIGN
	α1-3/4 fucosylation	T < S	RNA array: lower expression of *FUT4* and higher expression of *FUT11*<br>qPCR: higher expression of *FUT11*<br>MS: decreased abundance of total fucosylation in *N*-glycans, decreased abundance of α1-3/4 fucosylation in *O*-glycans, and depletion of α1-3/4 fucosylation in GSL-glycans<br>Binding assay: LTA and AAL
	Terminal GalNAc	T < S	MS: decreased abundance of terminal HexNAc in *N*-glycans, depletion of terminal GalNAc in *O*-glycans, and minor change in GSL-glycans<br>Binding assay: lower binding to SBA, WFA, HPA, and MGL
	Terminal GlcNAc	T < S	MS: decreased abundance of terminal HexNAc in *N*-glycans, depletion of terminal GlcNAc in *O*-glycans<br>Binding assay: lower binding to WGA
	Lewis A	T ≈ S	Binding assay: antibody

Glycan epitopes	Sialylated Lewis A	T < S	MS: decreased abundance in *O*-glycans<br>Binding assay: antibody
	Lewis B	T > S	Binding assay: antibody
	Lewis X	T < S	MS: depletion in *O*-glycans and GSL-glycans<br>Binding assay: antibody
	Sialylated Lewis X	T > S	Binding assay: antibody
	Lewis Y	T < S	Binding assay: antibody
	Blood group H	T < S	MS: Lower abundance in *O*-glycans<br>Binding assay: UEA-1
	Blood group A	T < S	MS: depletion in *O*-glycans and GSL-glycans<br>Binding assay: antibody
	Blood group B	T < S	Binding assay: antibody
	Fucosylated LacdiNAc	T < S	Binding assay: antibody

Note: >: Higher; ≈: Approxiamately equal; <: Lower; >>: significant higher; <<: significant higher; *: depleted expression; N.D.: not detected.
